# *MtING2* encodes an ING domain PHD finger protein which affects Medicago growth, flowering, global patterns of H3K4me3, and gene expression

**DOI:** 10.1111/tpj.15994

**Published:** 2022-10-17

**Authors:** Mauren Jaudal, Matthew Mayo‐Smith, Axel Poulet, Annabel Whibley, Yongyan Peng, Lulu Zhang, Geoffrey Thomson, Laura Trimborn, Yannick Jacob, Josien C. van Wolfswinkel, David C. Goldstone, Jiangqi Wen, Kirankumar S. Mysore, Joanna Putterill

**Affiliations:** ^1^ School of Biological Sciences University of Auckland Private Bag 92019 Auckland 1142 New Zealand; ^2^ Yale University Department of Molecular Cellular and Developmental Biology Faculty of Arts and Sciences 260 Whitney Avenue New Haven CT 06511 USA; ^3^ Institute for Plant Sciences, Biocenter University of Cologne Zülpicher Str. 47b 50674 Cologne Germany; ^4^ Institute for Agricultural Biosciences Oklahoma State University 3210 Sam Noble Parkway Ardmore OK 73401 USA

**Keywords:** flowering, vernalization, legume, Medicago, *MtING1*, *MtING2*, ING domain, PHD finger, epigenome reader, H3K4me3

## Abstract

Flowering of the reference legume *Medicago truncatula* is promoted by winter cold (vernalization) followed by long‐day photoperiods (VLD) similar to winter annual Arabidopsis. However, Medicago lacks *FLC* and *CO*, key regulators of Arabidopsis VLD flowering*.* Most plants have two *INHIBITOR OF GROWTH* (*ING*) genes (*ING1* and *ING2*), encoding proteins with an ING domain with two anti‐parallel alpha‐helices and a plant homeodomain (PHD) finger, but their genetic role has not been previously described*.* In Medicago, *Mting1* gene‐edited mutants developed and flowered normally, but an *Mting2‐1 Tnt1* insertion mutant and gene‐edited *Mting2* mutants had developmental abnormalities including delayed flowering particularly in VLD, compact architecture, abnormal leaves with extra leaflets but no trichomes, and smaller seeds and barrels. *Mting2* mutants had reduced expression of activators of flowering, including the *FT*‐like gene *MtFTa1*, and increased expression of the candidate repressor *MtTFL1c*, consistent with the delayed flowering of the mutant. *MtING2* overexpression complemented *Mting2‐1*, but did not accelerate flowering in wild type. The MtING2 PHD finger bound H3K4me2/3 peptides weakly *in vitro*, but analysis of gene‐edited mutants indicated that it was dispensable to MtING2 function in wild‐type plants. RNA sequencing experiments indicated that >7000 genes are mis‐expressed in the *Mting2‐1* mutant, consistent with its strong mutant phenotypes. Interestingly, ChIP‐seq analysis identified >5000 novel H3K4me3 locations in the genome of *Mting2‐1* mutants compared to wild type R108. Overall, our mutant study has uncovered an important physiological role of a plant *ING2* gene in development, flowering, and gene expression, which likely involves an epigenetic mechanism.

## Significance Statement

The timing of flowering is important for plant adaptation and crop productivity, but knowledge on how this is regulated in legumes is limited. Here, we report the functional characterization of *MtING2* and show that it is important in regulating plant architecture and flowering time in the model legume *Medicago truncatula*, providing evidence of the physiological role of an *ING‐like* gene in plants.

## INTRODUCTION

Legumes are the second most important group of crop plants after the cereals, but the rate of yield improvements in crop legumes overall lags well behind the cereals (Foyer et al., 2016). The timing of flowering is an important plant adaptive trait and key to crop productivity (Jung & Muller, 2009). *Medicago truncatula* (Medicago) and *Pisum sativum* (garden pea) are used as flowering time models for other temperate‐climate legume crops such as the fodder crops *Medicago sativa* (alfalfa) and *Trifolium* clovers, as well as pulse crops such as *Cicer arietinum* (chickpea) and *Lens culinaris* (lentil) (Benlloch et al., 2006; Tadege et al., 2009; Young et al., 2011). Medicago is an annual, diploid, transformable, self‐fertile plant that can be gene‐edited with genome sequences and mutant collections, making it of great use to study flowering time control (Jaudal, Thomson, et al., 2020; Meng et al., 2017; Tadege et al., 2009; Weller & Macknight, 2018; Young et al., 2011).

Medicago, like winter annual *Arabidopsis thaliana* (Arabidopsis), flowers in response to extended winter cold (vernalization) followed by warm, long‐day (LD) photoperiods in spring (VLD) (Clarkson & Russell, 1975). As in Arabidopsis and many other plants, the expression of a *FLOWERING LOCUS T‐LIKE* gene, *MtFTa1*, is elevated by these floral‐inductive signals in the leaves and has been shown to promote the transition to flowering in Medicago by both overexpression and mutant analyses (Jaudal, Thomson, et al., 2020; Laurie et al., 2011; Putterill & Varkonyi‐Gasic, 2016; Turck et al., 2008). However, interestingly, the direct upstream regulators of *MtFTa1* in Medicago and garden pea appear to differ from Arabidopsis. These temperate legumes lack a functional CONSTANS (CO), a crucial activator of Arabidopsis *FT* in the photoperiod pathway, and FLOWERING LOCUS C (FLC), a vernalization‐responsive repressor of *FT* in Arabidopsis (Jaudal, Thomson, et al., 2020; Kim & Sung, 2013; Weller & Macknight, 2018; Wong et al., 2014).

Arabidopsis *FT* encodes a florigen, which is activated by CO upon LD induction and moves from the leaves through the phloem to the shoot apex, where it interacts with the FLOWERING LOCUS D (FD) transcription factor and activates the expression of the MADS genes *SUPPRESSOR OF OVEREXPRESSION OF CONSTANS1* (*SOC1*) and *APETALA1* (*AP1*) to trigger flowering (Andres & Coupland, 2012; Putterill & Varkonyi‐Gasic, 2016; Zhu et al., 2021). To date, analysis of late‐flowering Medicago mutants indicates that *MtSOC1a* and *MtFDa* also promote the transition to flowering in Medicago (Cheng et al., 2020; Jaudal et al., 2018). Despite the lack of CO function, other components of the Arabidopsis photoperiod pathway such as the circadian clock, photoreceptors like PHYTOCHROME A (MtPHYA), activators such as MtFE, and repressors such as CYCLING DOF FACTORs (MtCDFs) regulate flowering in Medicago and/or garden pea, indicating that some aspects of the Arabidopsis photoperiodic regulatory network appear to be conserved in these plant species (Jaudal, Wen, et al., 2020; Kinoshita & Richter, 2020; Song et al., 2015; Thomson et al., 2021; Weller & Macknight, 2018; Weller & Ortega, 2015; Zhang et al., 2019).

In Arabidopsis, the MADS box transcription factor FLC is a major floral repressor and a target of the vernalization pathway. FLC represses the floral integrator genes *FT* and *SOC1* until being silenced by vernalization, thus allowing the CO‐mediated induction of *FT* and *SOC1* in the LD conditions of spring to promote flowering (Andres & Coupland, 2012). VERNALIZATION 2 (VRN2) is part of the polycomb repressive complex 2 (PRC2), which stably represses *FLC* expression (Andres & Coupland, 2012; Berry & Dean, 2015). Medicago lacks the FLC/MADS AFFECTING FLOWERING (MAF) clade of floral repressors, but has a *VRN2‐like* gene, *MtVRN2* (Jaudal et al., 2016). *MtVRN2* acts as a repressor of flowering by repressing *MtFTa1* prior to vernalization (Jaudal et al., 2016). This is in interesting contrast to the promotive role of VRN2 in flowering through its *FLC* repression in Arabidopsis (Gendall et al., 2001). Dominant early flowering Medicago *spring* mutants with retroelement insertions at the *MtFTa1* locus overexpress *MtFTa1* in LD conditions in the absence of vernalization, further supporting the idea that *MtFTa1* is repressed before vernalization in wild‐type plants (Jaudal et al., 2013; Yeoh et al., 2013).

*INHIBITOR OF GROWTH* (*ING*) genes were first named for the role of human *ING* genes as tumor suppressors (Doyon et al., 2006; Lee et al., 2009). The N‐terminal ING domains function in protein dimerization and interaction with other proteins in histone‐modifying complexes (Culurgioni et al., 2012; Ormaza et al., 2019; Palacios et al., 2010; Xu et al., 2016), while the plant homeodomain (PHD) zinc finger (Schindler et al., 1993) has been shown to bind H3K4me3 or H3K4me2 chromatin marks typically found near the transcription start site (TSS) in transcriptionally active genes (Peña et al., 2006; Roudier et al., 2011; Santos‐Rosa et al., 2002; You et al., 2017). ING proteins can regulate gene expression by recruiting chromatin‐activating or ‐silencing complexes (Jiang et al., 2020; Mouriz et al., 2015; Sanchez & Zhou, 2011; Shi et al., 2006). For example, human ING2 binds to H3K4me2/3 marks of its target genes, recruits HDACs, and is a stable subunit of the repressive mSin3a–HDAC1 histone deacetylase complex (Doyon et al., 2006; Peña et al., 2006; Shi et al., 2006).

In plants, PHD finger proteins are involved in a wide range of developmental and physiological processes, including flowering time control (Lee et al., 2009; Mouriz et al., 2015). However, to our knowledge, plant *ING* genes have not been characterized by mutation. In Arabidopsis, the two ING proteins (AtING1 and AtING2) form a phylogenetic subgroup amongst the 97 Arabidopsis PHD finger proteins (Alam et al., 2019). AtING proteins are nuclear‐localized in protoplasts and their PHD fingers bind H3K4me2 and H3K4me3 *in vitro* (Lee et al., 2009; Zhao et al., 2018).

Here, we report that in Medicago, *Mting1* gene‐edited mutants have a wild‐type appearance and flowering time. In contrast, using a Medicago *Tnt1* retroelement insertion mutant (*Mting2‐1*) and gene‐edited mutants including deletion mutants *Mting2‐2* and *Mting2‐3*, we show that Medicago plants carrying mutations in *MtING2* have strong mutant phenotypes. These include late flowering, especially under VLD conditions, small stature with altered leaf patterning and no trichomes, and small seed barrels and seeds compared to wild‐type R108 plants. The great majority of mutants with typical *Mting2* phenotypes carry mutations affecting the first alpha‐helix of the ING domain. While the MtING2 PHD finger weakly binds H3K4me2/3 *in vitro*, interestingly MtING2 PHD finger mutants grow and flower like wild‐type plants. RNA sequencing (RNA‐seq) indicates that >7000 genes are differentially expressed in the *Mting2‐1* mutant compared to wild type R108, including a broad range of candidate flowering‐related genes, which are consistent with its many abnormal phenotypes. Chromatin immunoprecipitation combined with sequencing (ChIP‐seq) analysis of the H3K4me3 mark indicates that the mutant has >5000 novel H3K4me3 locations compared with wild type R108. Overall, our study suggests that *MtING2* has an important, non‐redundant role in regulating Medicago growth, development, and flowering time as well as patterns of gene expression and H3K4me3.

## RESULTS

### *MtING1* and *MtING2* are broadly expressed and encode proteins with a predicted ING domain and a PHD finger

**Figure 1:**
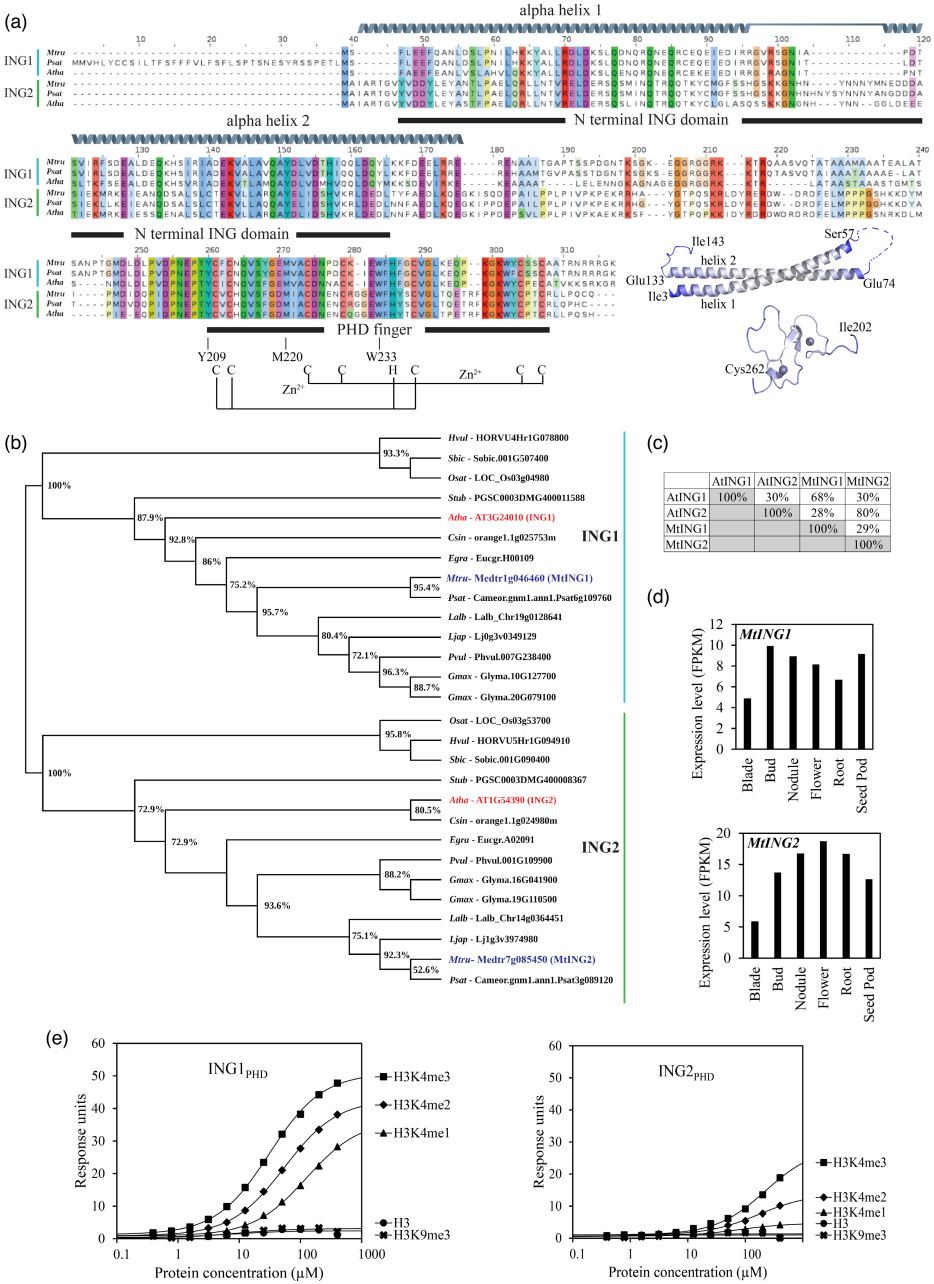
Features of the *MtING1* and *MtING2* genes and their predicted proteins. (a) Protein sequence alignment of Arabidopsis, Medicago, and garden pea INGs indicating the conserved N‐terminal ING domain and PHD finger as predicted by Pfam (Mistry et al., 2021) and SMART (Letunic et al., 2021). The ING domain is comprised of two alpha‐helices arranged in an anti‐parallel coiled coil. The MtING2 alpha‐helices (as predicted by AlphaFold) (Jumper et al., 2021) are shown as gray helices separated by a loop (H58–N73). Residues (Y209, M220, W233) within the PHD finger predicted to form an aromatic cage for recognition of H3K4me2/me3 and the C_3_H‐C_4_ residues that bind two zinc ions (Zn^2+^) to form a predicted zinc finger motif are shown (Peña et al., 2006). The AlphaFold models for the MtING2 ING domain and PHD finger are shown to the right of the alignment; residues with a confidence level of <50% have been excluded. The positions of the coordinated Zn^2+^ atoms are shown as spheres and two beta‐sheets are shown as arrows. (b) A consensus phylogenetic tree based on full‐length ING‐like proteins. The tree was generated using a maximum‐likelihood method with local support values based on Shimodaira–Hasegawa tests of 1000 resampled alignments. Nodes lacking annotation have less than 50% support. Sequences were drawn from Arabidopsis (Atha), *Citrus sinensis* (Csin), *Eucalyptus grandis* (Egra), *Glycine max* (Gmax), *Hordeum vulgare* (Hvul), *Lotus japonicus* (Ljap), *Lupinus albus* (Lalb), *Medicago truncatula* (Mtru), *Oryza sativa* (Osat), *Phaseolus vulgaris* (Pvul), garden pea (Psat), *Solanum tuberosum* (Stub), and *Sorghum bicolor* (Sbic). (c) Percentage identities between Arabidopsis and Medicago ING1 and ING2 proteins. (d) Gene expression of *MtING1* and *MtING2* in different tissues as determined by *in silico* analysis of normalized RNA‐seq data using the MedicMine *Medicago truncatula* Genome Database (Krishnakumar et al., 2015). (e) The PHD finger of MtING1 or MtING2 (ING_PHD_) and histone 3 peptides modified at lysine 4 (H3K4me1–3) were used to analyze the *in vitro* interaction. An unmodified peptide (H3) and H3K9me3 peptide were used as controls. Binding to peptides was determined by surface plasmon resonance.

*MtING1* (Medtr1g046460) and *MtING2* (Medtr7g085450) encode an N‐terminal ING domain comprised of two alpha‐helices arranged in an anti‐parallel coiled coil, a central disordered region, and a predicted C‐terminal Cys3‐His‐Cys4 PHD zinc finger (Figure 1a). The MtINGs have higher overall sequence identity with the corresponding Arabidopsis ING proteins than with each other, and thus group with either ING1‐like or ING2‐like proteins from other plants in a phylogenetic tree (Figure 1b,c). Most species in the tree have one of each ING protein (Lee et al., 2009), with the exception of the tropical legume *Glycine max* (soybean), which has two of each, likely the result of a soybean‐specific duplication (Shoemaker et al., 1996).

As reported for Arabidopsis *ING* genes (Lee et al., 2009), both Medicago *ING* genes are broadly expressed in different tissues (Figure 1d). Further analysis of *MtING2* indicates that it is also expressed consistently at similar levels throughout developmental time courses in leaves and shoot apices (Figure S1).

Since the MtING PHD fingers have the aromatic cage features for H3K4me3/2 recognition (Lee et al., 2009; Peña et al., 2006) (Figure 1a), we tested the interactions between MtING1_PHD_ or MtING2_PHD_ fingers and five H3 peptides *in vitro* using surface plasmon resonance (Figure 1e). MtING1_PHD_ preferentially bound to H3K4me3 over the di‐ and mono‐methylated peptides, with K_D_ values of 31 μm, 54.5 μm, and 125.5 μm, respectively. No interaction with H3K9me3 or the unmodified H3 peptide was observed (Figure 1e). However, the MtING2 PHD finger showed a much weaker interaction with H3K4‐methylated peptides. Although we were unable to determine a dissociation constant for these interactions, a preference for binding to H3K4me3 and H3K4me2 peptides was seen with limited binding detected with H3K4me1, and no binding to H3K9me3 or unmodified H3 (Figure 1e).

Arabidopsis HISTONE DEACETYLASE COMPLEX 1 (HDC1) interacts with AtING2 in bimolecular fluorescence complementation in tobacco (*Nicotiana benthamiana*) leaf nuclei. AtING2 also interacts with other proteins, including SIN3A ASSOCIATED PROTEIN 18 (SAP18) and SHORT LIFE 1 (SHL1), indicating that it may be part of a repressive chromatin complex (Lopez‐Gonzalez et al., 2014; Perrella et al., 2016). Here, we carried out yeast two‐hybrid assays (Figure S2) to test whether MtING1 and MtING2 interact with putative Medicago members of an HDAC complex regulating flowering time in Arabidopsis (Gu et al., 2013) including SAP18 (Perrella et al., 2016). However, no interactions were observed.

### The *MtING2 Tnt1* mutant *Mting2‐1* is compact and late‐flowering compared to *Mting1* gene‐edited mutants and wild type R108

To further analyze *MtING1*, we generated 12 independent *Mting1* mutants by *Agrobacterium*‐mediated CRISPR/Cas9 gene editing of wild‐type R108 leaf disks, using a construct with six guides (Figure S3, Tables S1, and S2). However, the mutants, including those homozygous for mutations at guide 1 causing a frameshift in the first alpha‐helix of the ING domain that affected the remainder of the protein or those with very premature stop codons or major deletions, resembled wild‐type plants in their growth and flowering time (Figure S3, Table S2). No *Tnt1 Mting1* mutant was present in the *Tnt1* collection (Tadege et al., 2008; Tadege et al., 2009).

**Figure 2:**
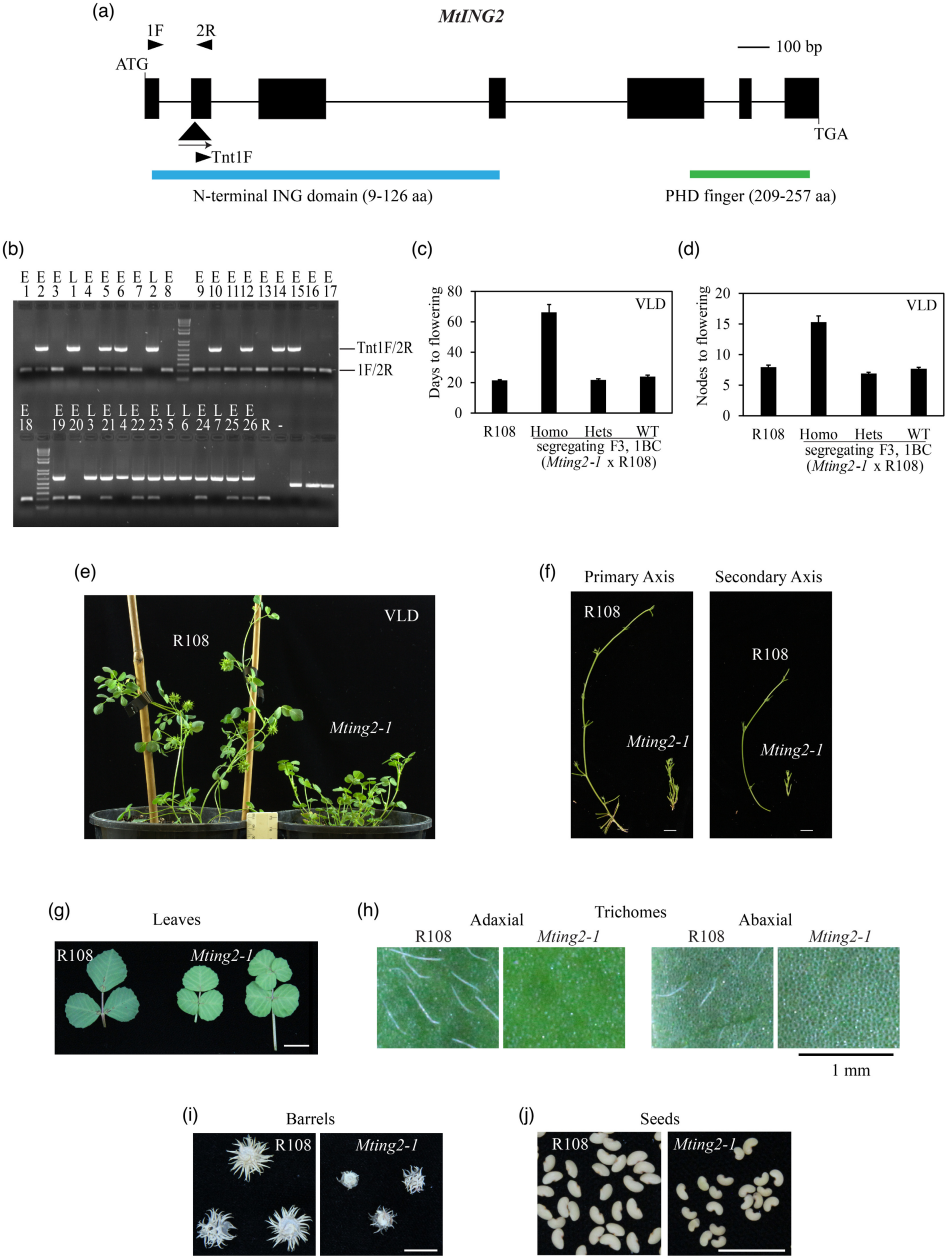
The *Mting2‐1 Tnt1* insertion mutant flowers late and is compact compared to wild‐type plants. (a) Diagram of the *MtING2* gene with the *Tnt1* insertion (black triangle) in the *Mting2‐1* mutant. Exons are shown as black boxes and introns as thin lines. Arrowheads indicate primers. (b) PCR genotyping fragments from a segregating F2, 1BC population of *Mting2‐1* × R108. Plants were scored as early‐ (E) (like R108) or late‐ (L) flowering relative to R108 in vernalized long‐day (VLD) conditions. 1F and 2R amplified a wild‐type band and Tnt1F and 2R amplified the *Tnt1* insertion. (c,d) Flowering time in VLD conditions scored as days to flowering (c) or the number of nodes on the primary axis at flowering (d) of R108 (*n* = 25) and a segregating F3, 1BC population of *Mting2‐1* × R108 (*n* = 160: *Mting2‐1 Tnt1* homozygotes, *n* = 21; heterozygotes, *n* = 74; wild‐type segregants, *n* = 65). Data are presented as mean ± 95% confidence interval. (e–h) Photographs of wild type R108 and *Mting2‐1* mutants showing 45‐day‐old plants grown in VLD conditions (e), primary and secondary axes (f), leaves including small or pentafoliate leaves from *Mting2‐1* (g), and trichomes on adaxial and abaxial sides of leaves, largely absent from *Mting2‐1* mutant leaves (h). (i,j) *Mting2‐1* plants have smaller seed barrels (i) and seeds (j) than wild type R108. Scale bar in (f,g,i,j) = 1 cm; scale bar in (h) = 1 mm.

For investigation of *MtING2*, an *Mting2 Tnt1* insertion mutant was identified through a flowering time screen and thus analysis was initially pursued with that mutant. Line NF1633 had a *Tnt1* insertion in exon 2 (Figure 2a, Figure S4). We named this mutant *Mting2‐1*. Line NF1633 segregated small plants with shorter primary and secondary axes (Figure 2e,f). These were late‐flowering compared to wild type R108 in flowering‐promotive VLD conditions (Figure 2e) and were homozygous for the *Tnt1* insertion in *MtING2*. The mutants had additional morphological phenotypes including abnormal leaves that were smaller (Figure 2g) and paler‐colored with no trichomes compared with wild type (Figure 2h). *Mting2‐1* leaves were often abnormal in pattern, with four or five leaflets, rather than typical trifoliate leaves (Figure 2g, Figure 5i). Mutant plants also produced smaller seed barrels with a lower number of seeds that were smaller than R108 (Figure 2i,j).

Next, we generated a segregating F2 population of 160 plants by crossing *Mting2‐1* with wild‐type R108 plants. All 21 *Mting2‐1* homozygotes recovered were late‐flowering (Figure 2b) and small in stature in VLD compared with wild type R108 (Figure 2e). The recessive, late‐flowering phenotype of *Mting2‐1* was seen in the next‐generation F3 population in both flowering time measures, days to flowering (Figure 2c) and number of nodes on the primary axis at the time of flowering (Figure 2d). The remaining heterozygous and wild‐type segregants flowered early like wild type R108 (Figure 2b–d) and were also similar in size to wild type. This indicated that the late‐flowering and compact architecture phenotypes showed 100% co‐segregation with the homozygous *Tnt1* insertion in *Mting2‐1*. These phenotypes were recessive and closely linked with the *Tnt1* insertion (within approximately 2.4 cM). Another striking feature of the segregating line was that far fewer (21 plants) homozygous mutant plants were recovered than the 25% expected (40 plants).

Thus, overall, *Mting2‐1* mutants showed a broad range of developmental defects, including compact architecture, delayed flowering time, and leaf, barrel, and seed abnormalities.

### *Mting2* gene‐edited deletion mutants *Mting2‐2* and *2‐3* are small, late‐flowering plants similar to *Mting2‐1*

**Figure 3:**
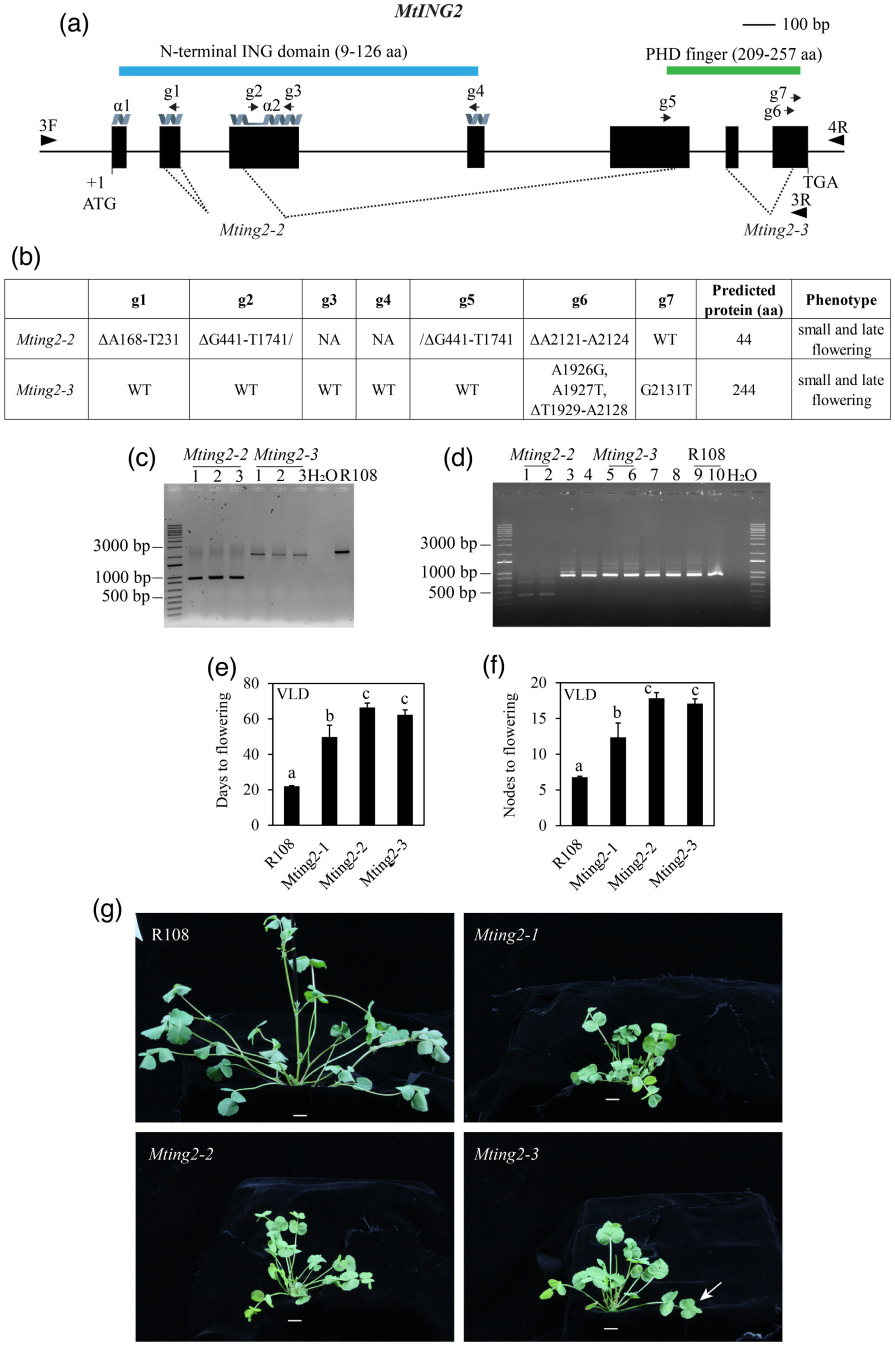
Plants carrying deletion mutant alleles *Mting2‐2* and *Mting2‐3* generated by CRISPR/Cas9 gene editing are small and late‐flowering similar to *Mting2‐1*. (a) Diagram of the *MtING2* gene showing the guides (g1–g7) used in CRISPR/Cas9 editing (arrows), the primers used in gDNA and cDNA amplification of edited lines (3F and 3R or 4R, arrowheads), and the gene‐edited mutant alleles (*Mting2‐2* and *Mting2‐3*) showing the major deletions in the genomic regions (dotted lines). Exons are shown as black boxes, introns are shown as thin lines, and the two alpha‐helices (α1 and α2) of the ING domain separated by a loop are indicated as gray helices. (b) Table summarizing the specific mutations and phenotype observed. (c) Photograph showing PCR genotyping fragments from homozygous *Mting2‐2* and *Mting2‐3* T1 plants compared with wild type R108 using primers 3F and 3R or 4R. (d) Photograph showing cDNA fragments from plants segregating in the T1 population (1–10) with *Mting2* mutant (*Mting2‐2* and *Mting2‐3*) or wild‐type‐like phenotypes (lanes 3, 4, 7, and 8) compared with R108 (lanes 9 and 10) using primers 3F and 3R. (e,f) Flowering time in VLD conditions scored as days to flowering (e) or the number of nodes on the primary axis at flowering (f) of R108 (*n* = 33) and homozygous *Mting2* mutants: *Mting2‐1* (F7, 2.5BC, *n* = 15), *Mting2‐2* (T2, *n* = 20), and *Mting2‐3* (T2, *n* = 16). Data are presented as mean ± 95% confidence interval. (g) Photographs of 27‐day‐old R108, *Mting2‐1* mutant (F5, 2.5BC), T1 *Mting2‐2* mutant, and T1 *Mting2‐3* mutant plants in VLD conditions. The arrow indicates a pentafoliate leaf. Scale = 1 cm.

To further confirm that mutations in *MtING2* led to late flowering and developmental defects, we carried out gene editing to obtain additional mutant alleles. A CRISPR/Cas9 construct with seven guides (g1 to g7) (Table S1) targeting *MtING2* (Figure 3a) was used. PCR genotyping indicated that several T1 lines had mutations in the *MtING2* gene (Table S2). These included two lines homozygous for genomic deletions, as shown by PCR fragments that were smaller in size compared with the wild‐type gene (Figure 3a–c). Next, we analyzed *MtING2* cDNA from these two T1 lines and wild type. The expected cDNA of approximately 900 bp was PCR‐amplified in wild‐type R108 plants (Figure 3d). However, *Mting2‐2* homozygous mutants produced only small, weak bands compared with wild type. The smallest and brightest band was approximately 500 bp, consistent with the deletions in the genomic DNA (Figure 3d). Its reduced abundance was consistent with the very premature stop codon in this allele. Direct sequencing of the cDNA PCR products indicated that the predicted protein is only 44 amino acids long (Figure 3b, Table S2) compared to 263 amino acids in wild type.

*Mting2‐3* homozygous plants, on the other hand, produced a similar‐length cDNA PCR product with similar intensity to wild type R108 (Figure 3d). This was unexpected due to the approximately 300‐bp deletions in the genomic DNA. Direct sequencing of the PCR product predicted a truncated protein of 244 amino acids long. The first 215 amino acids were identical to wild type R108, but a frameshift occurred at S216M, with alternative splicing leading to inclusion of intron 5 in the transcript, followed by a stop codon at position 245, resulting in an altered C‐terminus and a predicted disrupted PHD finger (Table S2).

Consistent with the predicted strong effects of the gene editing, plants homozygous for *Mting2‐2* and *Mting2‐3* were very similar to the *Mting2‐1 Tnt1* insertion mutant in appearance (Figure 3g). Analysis of the flowering time of *Mting2‐2* and *Mting2‐3* homozygotes in VLD indicated that they were also significantly later‐flowering in VLD conditions than wild‐type R108 plants (Figure 3e,f) and produced leaves with abnormal numbers of leaflets (Figure 3g, Figure 5i) with no trichomes and small seed barrels similar to *Mting2‐1*.

Thus, in summary, the results of our analysis of the *MtING2* gene‐edited deletion mutants (*Mting2‐2* and *2‐3*) and the *Tnt1* insertion allele (*Mting2‐1*) strongly support the suggestion that defects in *MtING2* lead to aberrant development including delayed flowering time in Medicago.

### Assessment of additional *Mting2* gene‐edited plants and predicted effects on the ING domain and PHD finger

We also determined *MtING2* genomic and cDNA sequences and the phenotype of 15 other gene‐edited plants, along with *Mting2‐2* and *2‐3* (Table S2). Out of a total of 17 gene‐edited lines, eight had typical *Mting2* mutant phenotypes, including compact architecture and delayed flowering. The majority of these (six out of eight) (*Mting2‐2* and *Mting2‐4* to *‐8*) were mutated at guide 1 in the coding sequence (CDS) encoding the first alpha‐helix of the ING domain, but also at guide 2 and other guides causing a frameshift and premature truncation or large deletion in the MtING2 protein (Figure 3a, Table S2). The seventh line (*Mting2‐3*) had an intact ING domain, but altered splicing led to inclusion of intron 5, altering the protein sequence from amino acid residue 216 onwards and possibly affecting protein folding or stability. Line 8 (*Mting2‐10*) had an unresolved structural rearrangement in *MtING2* and could not be amplified by PCR.

The ninth line (*Mting2‐9*) (Table S2) had a weaker phenotype, intermediate between wild type and typical *Mting2* mutants (modestly delayed flowering, smaller plants, seeds, and barrels, some abnormal leaf development, reduced trichomes). This mutant had an in‐frame deletion of four amino acids in the ING domain (deletion of K61–N64) due to a mutation at guide 2 and a disrupted PHD finger due to a frameshift (Table S2). However, this ING domain deletion affected part of the region that forms a loop (H58–N73) between the two anti‐parallel helices of the ING domain (Figure 1a, Figure 3a), and thus is predicted to be unlikely to disrupt ING function.

The remaining eight lines (*Mting2‐11* to *‐18*) developed and flowered like wild type (Table S2). Five were homozygous for small in‐frame deletions of one to seven amino acids (S60–Y67) within the MtING2 loop due to mutations at guide 2 (Figure 3a) similar to *Mting2‐9* above. However, interestingly, all eight lines had mutations predicted to strongly affect the PHD finger. This included four lines (*Mting2‐11*, *‐13*, *‐16*, and *‐18*) with a frameshift mutation before the PHD finger. The fifth mutant had a disrupted C_3_H‐C_4_ zinc‐binding motif (*Mting2‐17*). Two lines (*Mting2‐14* and *Mting2‐15*) (Table S2) had a loss of 60% of the PHD finger including E232 and W233, residues predicted to be essential for binding of H3K4me3 (Peña et al., 2006). Interestingly, the eighth line (*Mting2‐16*) expressed an alternatively spliced mRNA with the inclusion of part of exon 6, but a disrupted PHD finger, similar to *Mting2‐3*. However, unlike *Mting2‐3*, *Mting2‐16* contained an additional frameshift mutation leading to an upstream stop codon and truncation of the predicted protein.

Overall, this analysis indicates that part of the loop between the two alpha‐helices in the MtING2 ING domain can be deleted and the PHD finger can be deleted or perturbed, without affecting wild‐type‐like growth and flowering phenotypes. However, no wild‐type‐like plants with mutations at g1 (within the ING domain alpha‐helix 1) were obtained (Table S2). These results imply that the conserved ING alpha‐helices are likely important for MtING2 function in promoting growth and flowering in a wild‐type background.

### *Mting2‐1* flowers later than wild type with a strongly impaired response to vernalization in LD

**Figure 4:**
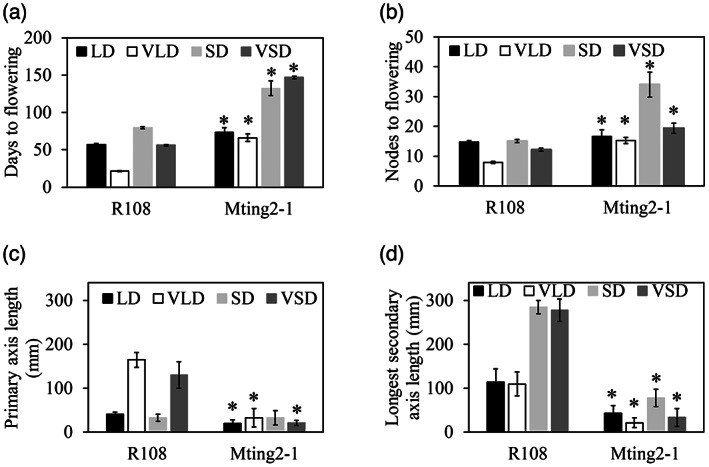
*Mting2‐1* causes late flowering with a strongly impaired response to VLD and decreased axis length. (a,b). Flowering time in different daylengths with and without prior vernalization (LD, VLD, SD, and VSD), scored as days to flowering (a) or the number of nodes on the primary axis at flowering (b) of wild type R108 and *Mting2‐1* mutants. Data are presented as mean ± 95% confidence interval (*n* = 6–25). (c,d) Primary axis (c) and secondary axis (d) measurements of R108 and *Mting2‐1* mutants in LD, VLD, SD, and VSD conditions. Data are presented as mean ± 95% confidence interval (*n* = 7–16). Asterisks (*) indicate significant differences from wild type R108, as determined using one‐way analysis of variance (α = 0.05).

To investigate the effect of the *Mting2‐1* mutation on growth and flowering in different environmental conditions, the mutants were grown in two photoperiods (LD or short days [SD]), with and without prior vernalization (Figure 4). As expected, wild type R108 in floral‐inductive VLD conditions flowered much more rapidly than in any other conditions (LD, SD, and VSD). In contrast, the *Mting2‐1* mutant showed compact growth and delayed flowering, to varying extents, in all the conditions compared with wild‐type R108 plants (Figure 4a,b).

One striking feature of the *Mting2‐1* mutant was that it was strongly impaired in its response to vernalization compared with wild‐type R108 plants in VLD conditions. Wild‐type plants flower in half the time or less in VLD compared to LD conditions (Figure 4a,b). However, the *Mting2‐1* mutants flowered at a similar time in VLD and LD, both slightly later than wild type in LD (Figure 4a,b). This indicates that *Mting2‐1* flowering was not promoted by vernalization in LD conditions, unlike wild‐type R108 plants. The *Mting2‐1* mutant also showed significantly decreased primary and secondary axis elongation in all conditions tested except for primary axis length in SD conditions (Figure 4c,d).

### Overexpression of *MtING2* does not accelerate flowering in wild‐type Medicago or Arabidopsis plants but complements the *Mting2‐1* mutant

**Figure 5:**
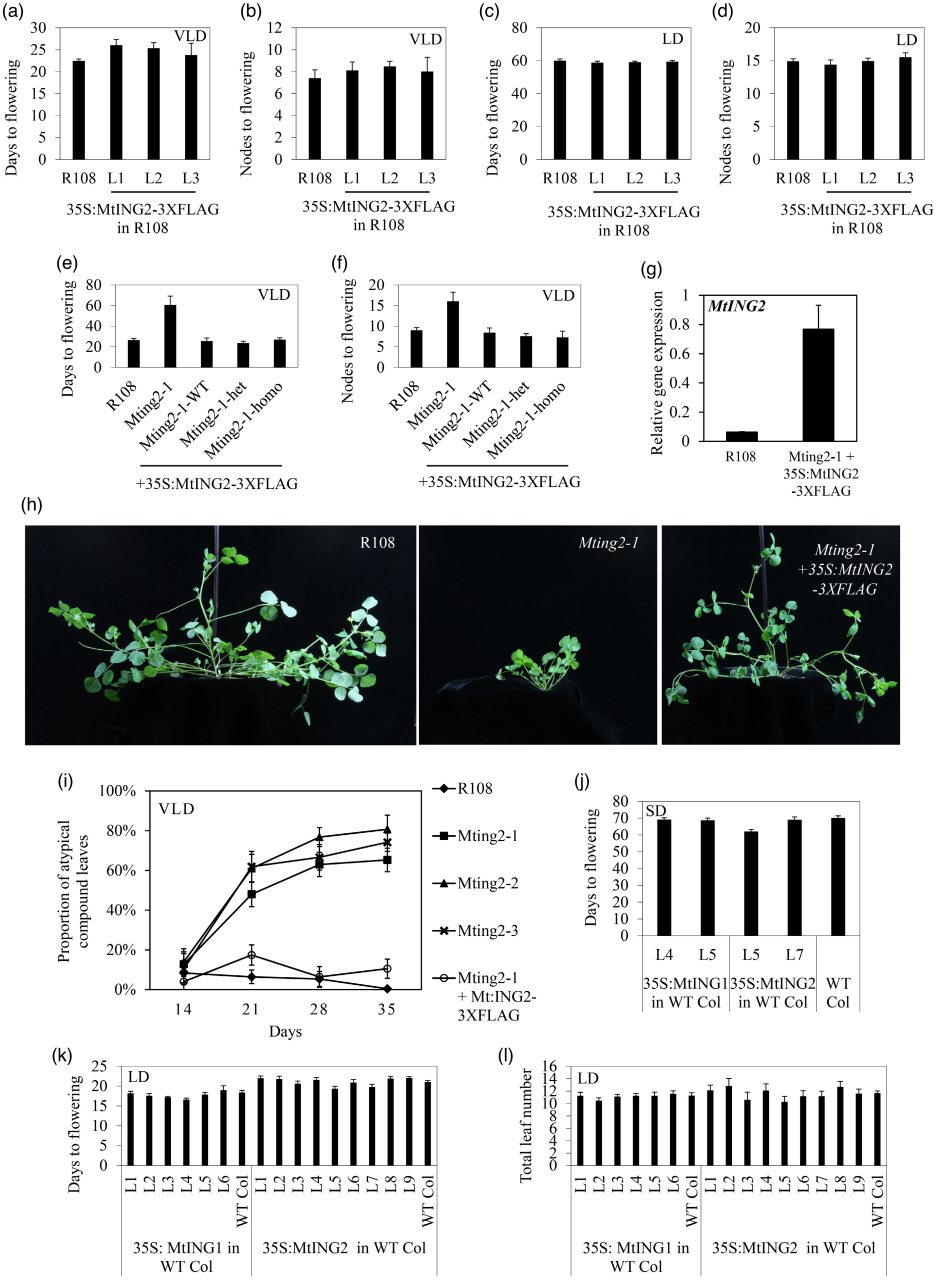
Overexpression of *MtING2* does not accelerate flowering in wild‐type Medicago or Arabidopsis but can restore the stature and flowering time of the *Mting2‐1* mutant. (a–d) Flowering time in VLD (a,b) and LD (c,d) conditions of three independent transgenic *35S:MtING2‐3xFLAG* lines (L1 to L3) in an R108 background scored as days to flowering (a,c) or as the number of nodes on the primary axis at flowering (b,d). Data are presented as mean ± 95% confidence interval (*n* = 10–45). T1 plants were used in (a,b), T1 and T2 plants were used in (c,d). (e,f) Flowering time of the segregating T2 progeny of a transformant with *35S:MtING2‐3xFLAG* in the *Mting2‐1* background in VLD conditions. Flowering time was scored as days to flowering (e) or the number of nodes on the primary axis at flowering (f) of R108 (*n* = 9), the *Mting2‐1* mutant (F5, 2.5BC) (*n* = 8), and segregating T2 progeny with the *35S:MtING2‐3xFLAG* transgene (*Mting2‐1 Tnt1* homozygotes, *n* = 4; heterozygotes, *n* = 11; wild‐type segregants, *n* = 8). Data are presented as mean ± 95% confidence interval. (g) Expression of *MtING2* in the leaves of 14‐day‐old *Mting2‐1* + *35S:MtING2‐3XFLAG* relative to R108. Plants were grown in VLD conditions and harvested at 4 h after dawn. *MtING2* expression was normalized to *PP2A*. Data are presented as mean ± 95% confidence interval of three biological replicates, with one plant per replicate. (h) Photographs of 34‐day‐old wild type R108, the *Mting2‐1* (F5, 2.5BC) mutant, and a rescued *Mting2‐1* plant (with *35S:MtING2‐3XFLAG*) in VLD conditions. (i) Proportion of atypical compound leaves (four or five leaflets, rather than three) in *Mting2* mutants compared with wild type R108 and *Mting2‐1* + *35S:MtING2‐3xFLAG* in the first 35 days of growth (*n* > 10 plants). (j–l) Flowering time of Arabidopsis transgenic lines with either the *35S:MtING1* or *35S:MtING2* transgenes in SD (8 h light, 16 h dark) (j) or LD (16 h light, 8 h dark) (k,l) photoperiods in the Columbia (Col) background. Flowering time was measured as either the total days to flowering (j,k) or the total number of rosette and cauline leaves at flowering (l). Data are presented as mean ± 95% confidence interval (*n* = 10–45). T3 plants were used in (j) and T2 plants were used in (k,l).

To test if overexpression of *MtING2* accelerates Medicago flowering, a 35S construct (*35S:MtING2‐3xFLAG*) was introduced into wild type R108, and transgenic T0 plants were selected. However, analysis of three independent T1 lines indicated that they flowered at a similar time to wild‐type R108 controls in both VLD and LD conditions (Figure 5a–d). The *35S:MtING2‐3xFLAG* gene was confirmed to be overexpressed in these lines by quantitative reverse transcriptase PCR (qRT‐PCR) (Figure S5). Western blot analysis using an anti‐FLAG antibody also indicated that a protein of the expected size was produced in the transgenic plants (Figure S5). In addition, in a complementation experiment (Figure S5), the delayed flowering, small plant size phenotype, and aberrant compound leaf pattern of the *Mting2‐1* mutant were rescued by transformation with the *FLAG* construct (Figure 5e–i).

We also transformed *35S:MtING1* and *35S:MtING2* gene constructs into Arabidopsis Columbia (Col) wild‐type plants (Figure 5j–l). However, transformants flowered at a similar time to wild type in LD (Figure 5k,l) or SD conditions (Figure 5j).

### A large number of genes are differentially expressed in the *Mting2‐1* mutant with partial overlap with mis‐regulated genes in other late‐flowering Medicago mutants

**Figure 6:**
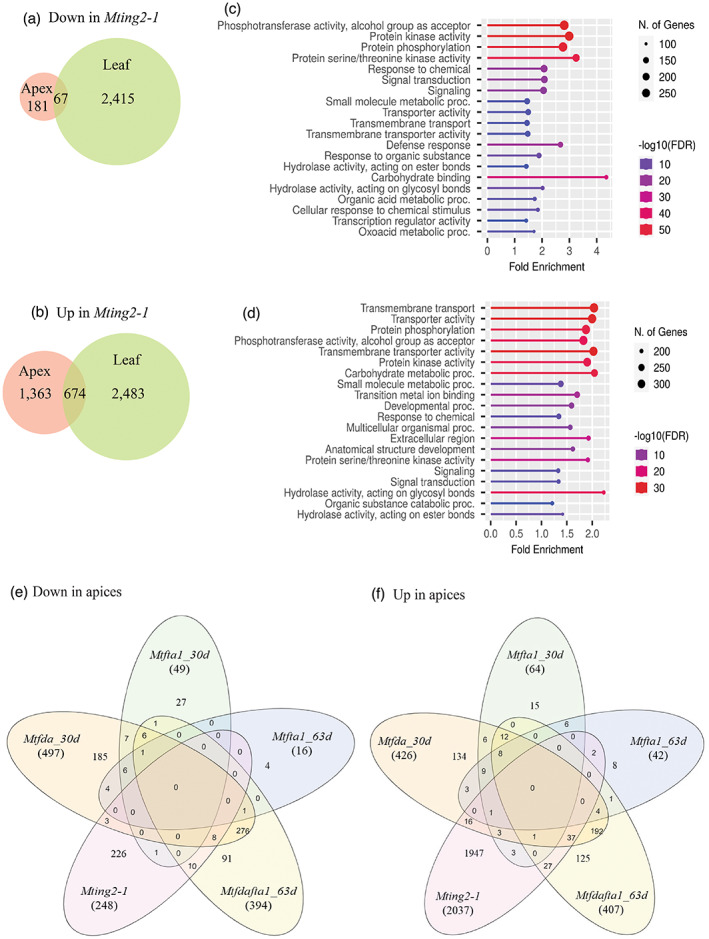
Identification of DEGs in the *Mting2‐1* mutant by RNA‐seq and comparison with other late‐flowering Medicago mutants. (a,b) Venn diagrams showing the number of DEGs with either decreased (a) (down) or elevated (b) (up) expression in the leaf and shoot apex of *Mting2‐1* mutants compared with wild type R108 as determined by RNA‐seq (¦log_2_(fold change)¦ ≥ 1 and adjusted *P*‐value ≤ 0.05). (c,d) Graphs showing enriched gene ontologies/functional classification of genes with either decreased (c) or elevated (d) transcript levels in *Mting2‐1* mutants (both leaf and apex) versus wild type. Overrepresented genes were classified based on GO biological processes, molecular functions, and cellular components (FDR < 0.05) in ShinyGO 0.76 (Ge et al., 2020). Homozygous *Mting2‐1* mutants were selected after two backcrosses to wild type R108. Three biological replicates per genotype were harvested 4 h after dawn and tissues were obtained from three‐leaf stage plants (approximately 13–15 days old) in VLD conditions. (e,f) Venn diagrams showing the number of genes either down (e) or up (f) in the *Mting2‐1* apex compared with data of the DEGs relative to segregating wild‐type‐like flowering plants (30 days old) in the shoot apices of late‐flowering Medicago mutants at the vegetative stage: *Mtfda_30d* (30 days old), *Mtfta1_30d* (30 days old), *Mtfta1_63d* (63 days old), and the *Mtfdafta1_63d* double mutant (63 days old), from Cheng et al. (2020), retrieved from https://www.ncbi.nlm.nih.gov/bioproject/PRJNA649443.

To further investigate the molecular basis of *Mting2‐1* mutant phenotypes, we carried out RNA‐seq analysis on leaves and shoot apices of 13–15‐day‐old *Mting2‐1* mutant and wild‐type R108 plants grown in VLD conditions in agar tubs. A large number of genes (7183, which represents 12.6% of the total number of protein‐coding genes in Medicago genome v4.0) were differentially expressed in the mutant relative to wild type in leaves and/or apices using the criteria of ¦log_2_(fold change)¦ ≥ 1 and adjusted *P*‐value ≤ 0.05 (Figure 6a,b, Table S3, Figure S6).

Amongst the differentially expressed genes (DEGs), more genes (4520) showed elevated expression (up) compared to reduced expression (down) (2663) in the *Mting2‐1* mutant relative to wild type R108 (Figure 6a,b). In the apex this difference was increased, with an eight‐fold increase in up genes compared to down genes in the *Mting2‐1* mutant (Figure 6b). Within the down category only 9% were differentially expressed in the apex and the remainder were in the leaves (Figure 6a). Gene Ontology (GO) analysis (Table S4) indicated significantly enriched categories such as protein phosphorylation, signaling, transporter activity, and carbohydrate metabolic processes (Figure 6c,d, Table S4).

Lists of candidate flowering time‐ and/or flowering‐related genes or their homologs were presented in previous reports (Cheng et al., 2020; Thomson et al., 2019; Zhou et al., 2021). Using these, within our *Mting2‐1* DEGs, we identified 110 candidates (Table S3); 35 genes encode MADS transcription factors, 36 genes are implicated in the photoperiod pathway, four are implicated in the age‐related pathway, one is implicated in carbohydrate status, four are implicated in vernalization, 19 are implicated in gibberellin hormone‐related processes, two encode TERMINAL FLOWER 1 (TFL)‐like/BFT, and nine are chromatin‐remodeling associated genes. There were also seven genes encoding histone methyltransferases and two genes encoding jumonji domain‐containing demethylases that were differentially expressed (Table S3). We also identified genes encoding different classes of transcription factors, some of which are implicated in flowering, annotated as MYB‐LIKE (79 genes), TCP‐LIKE (eight), and WRKY‐LIKE (39) (Table S3), and candidate stress‐related proteins (184) (Zhou et al., 2021) (Table S3).

Amongst the candidate flowering‐related genes, interestingly, the genes encoding candidate flowering repressors MtTFL1c and MtBFT and one of the three Medicago SHORT VEGETATIVE PHASE (MtSVP)‐like MADS transcription factors were up in the apices of *Mting2‐1* relative to wild type R108, consistent with the delayed flowering of the mutant (Table S3). Amongst the *FT‐like* genes, *MtFTa2* expression was significantly reduced in *Mting2‐1* (Table S3). *MtFTa2* was previously implicated in the vernalization pathway because its expression is promoted by vernalization (Laurie et al., 2011), but it has not been functionally characterized by mutation (Thomson et al., 2021). Possible promoters of flowering, the MADS transcription factor genes *MtAP1b*, *MtFRUITFULLb* (*MtFULb*), and *MtSOC1c*, were reduced in expression in the *Mting2‐1* mutant (Table S3).

The expression of genes encoding gibberellin 2 beta‐dioxygenases, usually associated with inactivation of bioactive gibberellins, was mostly elevated in the mutant compared with wild type R108 (Table S3), which may contribute to the small stature of the mutant plants (Hedden & Sponsel, 2015).

The DEGs in the shoot apices of the *Mting2‐1* mutant were also compared with those observed by RNA‐seq in the shoot apices of other late‐flowering Medicago mutants (*Mtfda*, *Mtfta1*, and *Mtfdafta1*) at vegetative stages (30 days and/or 63 days old) (Cheng et al., 2020) (Figure 6e,f, Table S3). The great majority of the *Mting2‐1* DEGs were unique to it (Figure 6e,f). However, out of 248 DEGs down in *Mting2‐1* shoot apices, approximately 9% (22 genes) were also down in the shoot apices of at least one of the other Medicago mutants (Figure 6e) (Cheng et al., 2020). These included the putative floral‐promotive genes *MtAP1b* and *MtSOC1c*, which had significantly reduced expression in the apices of *Mting2‐1* and were also down in the shoot apices of both *Mtfda* and *Mtfta1* at 30 days old (Table S3). Amongst the 2037 DEGs up in the apex of *Mting2‐1*, approximately 4% (90 genes) were also up in at least one of the other Medicago flowering mutants (Figure 6f). Within these, the putative floral repressor gene *MtSVP‐like* was up in the apices of *Mting2‐1* and in the apices of *Mtfda* at 30 days old and in *Mtfta1* at both 30 and 63 days old (Table S3).

**Figure 7:**
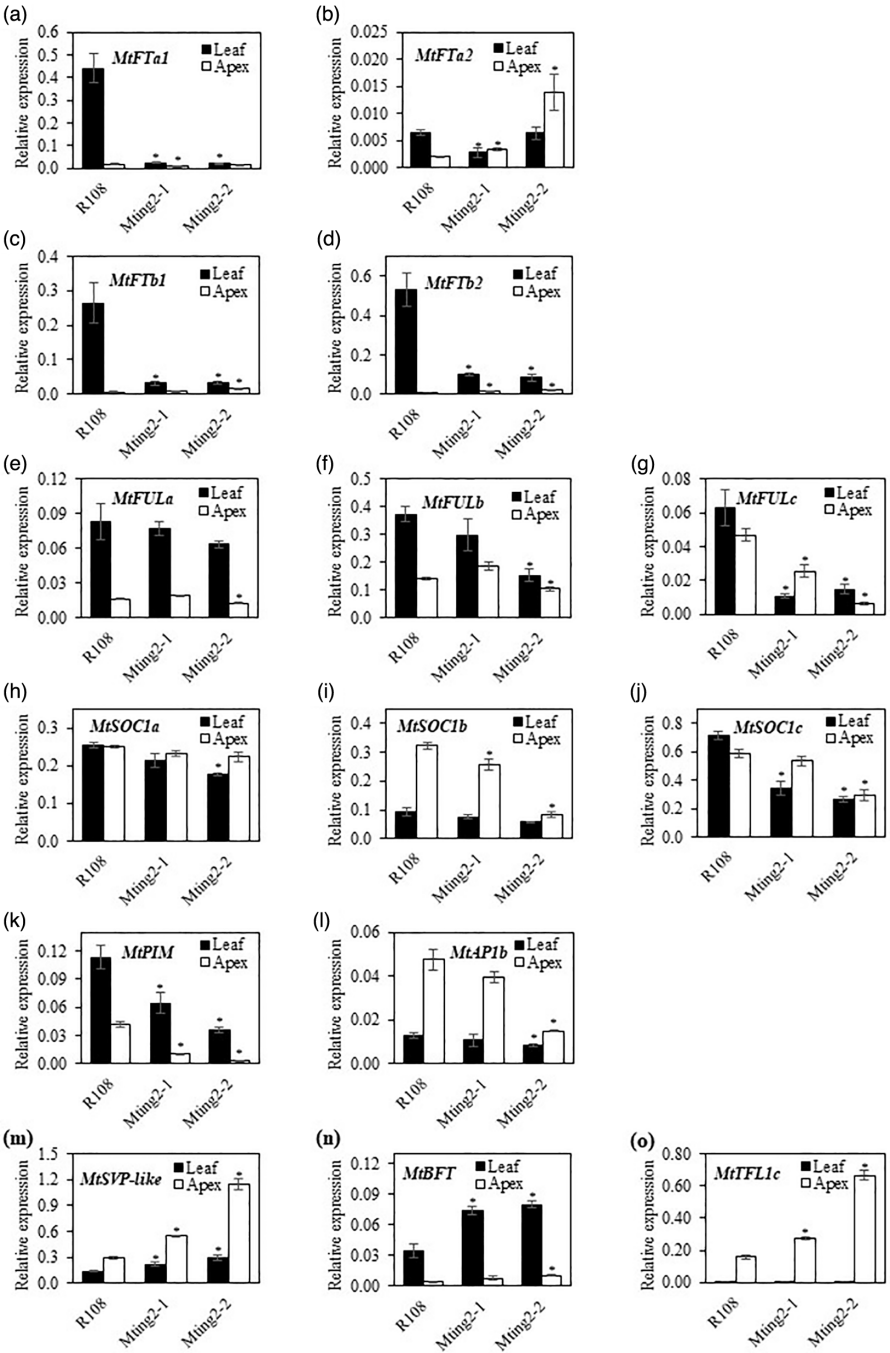
Analysis of expression of selected candidate floral activators and repressors. Relative gene expression measured by qRT‐PCR of leaf and apex samples of 14‐day‐old R108 and 16‐day‐old *Mting2‐1* and *Mting2‐2* plants grown in VLD conditions in soil pots. Mutant tissues were harvested 2 days later than wild type R108 to account for developmental differences caused by slower growth of the mutants. Tissues were harvested 4 h after dawn. Gene expression was calculated using the 2^−ΔΔCt^ method, where ΔCt was obtained by normalizing the expression level of the gene of interest to that of the reference gene, *PP2A*. Data are presented as mean ± SE of three biological replicates. Asterisks indicate significant differences in expression from wild type R108, as determined using one‐way analysis of variance (*α* = 0.05).

In an independent experiment on mutants (*Mting2‐1* and *Mting2‐2*) and wild‐type plants grown in VLD conditions in soil and analyzed by qRT‐PCR (Figure 7), amongst the *FT‐like* genes, the three LD‐induced, leaf‐expressed genes, *MtFTa1*, *MtFTb1*, and *MtFTb2* (Laurie et al., 2011), were most strikingly altered in the mutants (Figure 7a–d). All were significantly reduced in the leaves of *Mting2‐1* and *Mting2‐2* mutants compared to R108. Low levels of *MtFTa1* were consistent with the late flowering observed in the *Mtfta1* mutants in VLD conditions (Laurie et al., 2011). Of the three *MtFUL* genes (Figure 7e–g), *MtFULc* showed the strongest differential expression, being expressed at significantly lower levels in the leaves and apices of both *Mting2* mutants relative to R108 (Figure 7g). For *MtFULb*, *Mting2‐1* had slightly reduced expression in the leaves similar to RNA‐seq and *Mting2‐2* showed a moderate but significant reduction (Figure 7f). Of the three *MtSOC1* genes (Figure 7h–j), as observed in RNA‐seq, *MtSOC1c* expression was significantly lower in both *Mting2* mutants in the leaves compared to R108 (Figure 7j). For *MtSOC1b*, in the apex, expression was slightly (but significantly) lower in *Mting2‐1* compared to R108 with a strong, significant reduction in *Mting2‐2* (Figure 7i). *MtPIM* (an *AP1‐like* gene) expression was significantly lower in both *Mting2* mutants compared to R108, in leaves and apices, with *Mting2‐2* having the lowest expression in both tissue types, consistent with the delayed transition to flowering in the mutants (Figure 7k). *MtAP1b* expression in the apex was weakly reduced in *Mting2‐1*, similar to RNA‐seq, but significantly reduced in *Mting2‐2* compared with R108 (Figure 7l). The three candidate repressor genes, *MtSVP*, *MtBFT*, and *MtFTLc* (Figure 7m–o), showed a significant increase in expression in apices of both *Mting2* mutants relative to R108, as observed in the RNA‐seq results. *MtSVP‐like* and *MtBFT* expression was elevated in the leaves of the mutants, which was significantly different from R108.

### ChIP‐seq indicates new genomic regions enriched with H3K4me3 in the *Mting2‐1* mutant

The chromatin mark H3K4me3 is typically found at the 5′ end of active protein‐coding genes near the TSS. However, histone modification on chromatin is a dynamic process accompanied by changes in gene expression to regulate developmental changes. For example, in Arabidopsis, *FLC* expression is regulated by the dynamic H3K4me3 deposition by the PHD finger protein ARABIDOPSIS TRITHORAX1 (ATX1), a histone H3K4 methyltransferase which promotes and maintains the transcriptionally active state of *FLC* to repress the floral transition (Pien et al., 2008). In addition, for example, Miura et al. (2020) found that the resting level of H3K4me3 on the *WRKY70* gene was similarly high in stressed and non‐stressed *siz1* mutants, compared to its normal elevation only in stressed wild‐type plants. This result and others suggested that the PHD finger SUMO E3 ligase SIZ1 may work as a transcriptional repressor through binding with ATXs and with H3K4me3 (Miura et al., 2020).

**Figure 8:**
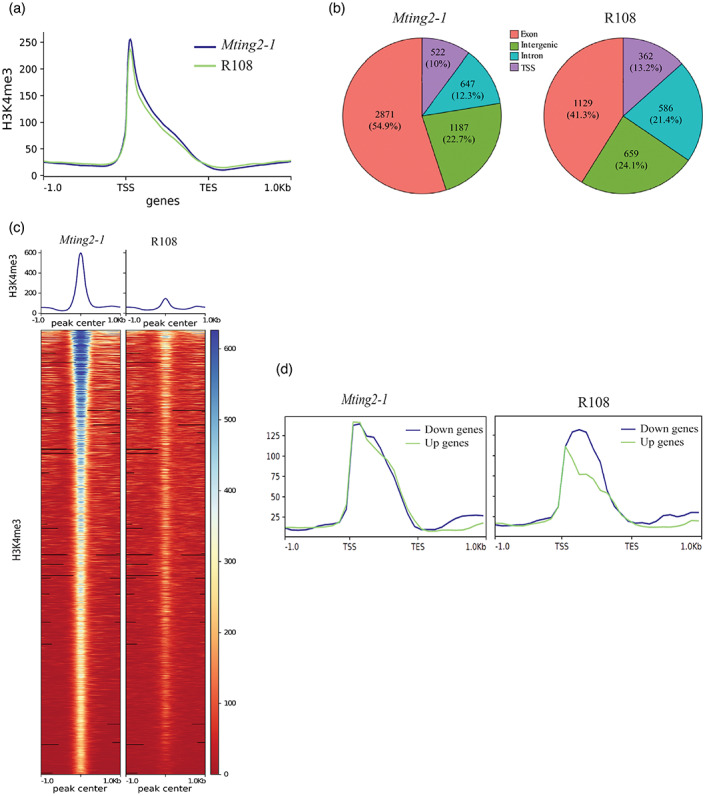
Analysis of H3K4me3 peaks in the *Mting2‐1* mutant compared to wild type R108. (a) Averaged normalized read counts of H3K4me3 over protein‐coding genes (± 1 kb) for the *Mting2‐1* mutant and wild type R108 in RPKM. TSS, transcription start site; TES, transcription end site. (b) Genome‐wide distribution of H3K4me3 in the *Mting2‐1* mutant and wild type R108 using two biological replicates. TSS, transcription start site. (c) Averaged normalized read counts and heatmap of H3K4me3 reads over peaks (± 1 kb) specific to the *Mting2‐1* mutant. (d) Average profile plot of H3K4me3 RPKM over the genic regions (± 1 kb) of genes with expression elevated (up, green line) and reduced (down, blue line) in the *Mting2‐1* mutant (merged apex and leaf RNA‐seq dataset) compared to wild type R108. The DEGs were selected based on adjusted *P*‐value < 0.05 and log_2_ (fold change) > 1 for up genes and log_2_(fold change) < −1 for down genes.

To investigate if the *Mting2‐1* mutation affected the pattern of H3K4me3, we carried out ChIP‐seq on the aerial parts of plants grown in agar tubs in VLD conditions (Figure 8). We saw an enrichment of H3K4me3 marks at the 5′ end of protein‐coding genes near the TSS in both *Mting2‐1* and wild type R108 as expected (Figure 8a). The distribution of H3K4me3 on the genomic regions was also similar overall between *Mting2‐1* and wild type R108 (Figure 8b) with the major proportion (>75%) of H3K4me3 peaks on the genic regions (TSSs, exons, and introns) in both genotypes and some peaks deposited on intergenic regions (Figure S7).

However, interestingly, we identified 5227 regions (including genic and intergenic regions) with H3K4me3 specifically in *Mting2‐1* (*P* < 0.05) (Figure 8c, Table S5, peaks specific to wild type R108 are shown in Figure S7). We observed an average of approximately six‐fold enrichment of H3K4me3 in *Mting2‐1* in these regions compared to wild type. We defined 3520 genic regions that overlapped 3375 genes with *Mting2‐1*‐specific H3K4me3 peaks (Figure S7). The genes with *Mting2‐1*‐specific H3K4me3 marks were predicted to be involved in a wide range of processes including molecular functions such as transcription regulator activity and enzyme regulator activity (Figure S7). *Mting2‐1* and wild‐type R108 plants were grown in agar tubs in an independent experiment and ChIP‐qPCR was carried out on selected genes (Figure S7).

Next, we tested if there was an impact of the novel regions of H3K4me3 in *Mting2‐1* aerial tissues on gene expression. To facilitate the comparison, we first merged the RNA‐seq datasets of apex and leaf for *Mting2‐1* compared to R108 as a proxy for aerial tissue data, and identified DEGs (Table S5). There were 3056 DEGs, with 1069 showing reduced expression (down) in *Mting2‐1* and 1987 showing elevated expression (up) in *Mting2‐1* relative to R108. We then compared the H3K4me3 signal around the genes that were up and down in the *Mting2‐1* mutant (Figure 8d, Table S5). We observed a slight enrichment of H3K4me3 on the genes that were up in *Mting2‐1* mutant compared to those in wild type in the merged data, whereas the H3K4me3 levels of the genes that were down were similar between *Mting2‐1* and wild type (Figure 8d). There were 343 DEGs in the merged datasets that were enriched with H3K4me3 marks, and approximately 70% of these showed elevated expression in the *Mting2‐1* mutant (Table S5). Amongst the candidate genes (Table S5), some stress‐related cytochrome P450 genes and genes encoding MYB and WRKY transcription factors that were up in the *Mting2‐1* mutant were also enriched with *Mting2‐1*‐specific H3K4me3 marks. A gene encoding a chromatin‐remodeling SWI/SNF complex component SNF12 homolog (Medtr3g089140) was up in the *Mting2‐1* mutant and enriched with the *Mting2‐1*‐specific H3K4me3 mark. Medtr3g117280, encoding a putative SHL, was not differentially expressed, but was marked with *Mting2‐1*‐specific H3K4me3.

Similar results were observed when the H3K4me3 signals were compared with DEGs in the apex or leaf (RNA‐seq data from Table S3) (Figure S8, Table S6). There was a slight enrichment of H3K4me3 on the genes that were up in the apex or leaf in the *Mting2‐1* mutant compared to the wild type R108. Taken together, these results suggest that the increased H3K4me3 marks may be correlated with elevated gene expression in *Mting2‐1*.

## DISCUSSION

### *Mting2* mutants are compact with developmental abnormalities and global changes in gene expression and H3K4me3

Here we identified and functionally characterized *MtING2*, encoding an ING domain PHD finger protein that influences plant growth, architecture, and flowering time control in Medicago. RNA‐seq and ChIP‐seq data from the *Mting2‐1* analysis suggest that a broad range of gene programs are altered in the *Mting2‐1* mutant compared to wild type, consistent with its many abnormal phenotypes. Amongst the total DEGs from RNA‐seq of leaves or shoot apices, nearly twice as many genes showed elevated expression compared to reduced expression in the *Mting2‐1* mutant relative to wild type R108. We also observed more than 5000 novel H3K4me3 *Mting2‐1*‐specific regions in aerial tissues that were not observed in wild type. The H3K4me3 marks in *Mting2‐1* overlapped with some of the genes that are up in *Mting2‐1*, suggesting a correlation in some cases with differentially elevated gene expression. These features of the *Mting2‐1* mutant may suggest a prominent role overall of MtING2 as a repressor of gene expression as previously observed for INGs from some other systems (Doyon et al., 2006; Peña et al., 2006; Shi et al., 2006).

### *Mting2* mutants flower late with a greatly reduced response to vernalization in LD conditions

While *Ating* mutant plants have not yet been reported, Arabidopsis plants with mutations in genes encoding other PHD finger proteins including *early bolting in short days* (*ebs*), *shl*, and *at‐rich interacting domain 5* (*arid5*) exhibited an array of developmental defects such as smaller plant size, smaller and fewer leaves, smaller siliques, and early flowering compared to the wild type (Gómez‐Mena et al., 2001; Lopez‐Gonzalez et al., 2014; Qian et al., 2018; Tan et al., 2020). Expression of the floral activators *FT* and *SOC1* was elevated in *shl* and *ebs* mutants, respectively (Lopez‐Gonzalez et al., 2014). In *arid5*, the expression of floral repressors *FLC* and *SVP* was reduced while the expression of floral activators *FT* and *SEP3* was elevated, consistent with the early flowering observed (Tan et al., 2020).

Medicago *Mting2* mutants are also small plants with developmental defects, but they are late‐flowering compared to wild type R108. The strongly impaired response of the *Mting2‐1* mutant to VLD is similar to the late‐flowering *Mtfta1* mutants (Jaudal, Thomson, et al., 2020; Laurie et al., 2011) and flowering delay in *Mting2* correlated with a reduction in *MtFTa1* expression compared to wild type. This indicates that MtING2 promotes *MtFTa1* expression, but it is not yet clear how directly this occurs. Amongst other *FT‐like* genes, *MtFTb1* and *MtFTb2* had significantly reduced expression in leaves of *Mting2* mutants compared with wild type, but have not been demonstrated to regulate flowering (Thomson et al., 2021). In addition, there was elevation in expression of candidate repressor PEBP genes including an *MtBFT‐like* gene in leaves and *MtTFL1c* in the mutant shoot apices compared to wild type R108. The garden pea homolog of *MtTFL1c* is *LATE FLOWERING* (*LF*), which represses flowering, with flowering time being very sensitive to the level of *LF* in the shoot apex (Foucher et al., 2003).

A MADS *MtSVP‐like* gene that was previously functionally uncharacterized (Jaudal et al., 2014) was upregulated in the *Mting2‐1* mutant and some other late‐flowering mutants and may function to repress flowering. Other potential activators of flowering in Medicago had reduced expression in the *Mting2‐1* mutant in VLD conditions, which may contribute to late flowering. For example, *MtFULb* and *MtSOC1c* expression was reduced in the *Mting2* mutants. These two MADS genes were also expressed at a lower level in the *Mtfta1* mutant than in wild type R108 (Jaudal et al., 2015; Jaudal et al., 2018) and in the late‐flowering *Mtphya‐1* mutant (Jaudal, Wen, et al., 2020). Their expression is promoted by VLD and they promote flowering in Arabidopsis (Fudge et al., 2018; Jaudal et al., 2015; Jaudal et al., 2018), but they have not yet been shown to affect flowering time in Medicago by mutation studies. The expression of a gene (Medtr1g098000) encoding a homolog of the Arabidopsis histone methyltransferase ASHH1/SDG26 was elevated in *Mting2‐1*. ASHH1/SDG26 binds to Arabidopsis *SOC1* and is required for H3K4me3 deposition on this locus to promote flowering (Berr et al., 2015). The RNA‐seq data also revealed several flowering time‐related gene candidates that are mis‐regulated in the *Mting2‐1* mutant compared with wild type R108, including genes that encode other MADS transcription factors, CCT domain proteins, and chromatin remodeling‐associated proteins, amongst others, which may also have a role in Medicago flowering time regulation and plant development.

### Role of the MtING2 ING domain and PHD finger

The majority of lines with typical *Mting2* mutant phenotypes had mutations that were predicted to perturb the first conserved alpha‐helix of the ING domain. On the other hand, some other ING domain mutants grew and flowered like wild type. The latter had small in‐frame mutations causing small deletions in the loop between the two ING alpha‐helices that are unlikely to affect ING domain function. There were also *Mting2* gene‐edited lines encoding altered or deleted PHD fingers that grew and flowered normally. Interestingly, work on *Fusarium* ING, FNG1, showed a similar result, indicating that the PHD finger of this ING protein does not appear to be essential for its *in vivo* function (Jiang et al., 2020). Overall, the results indicate that the conserved ING domain alpha‐helices may have an important non‐redundant function in MtING2‐promoted flowering and development. To confirm this, the role of the different domains of MtING2 should be further tested by complementation experiments with different regions of the MtING2 gene and/or constructs carrying specific mutations in either the ING domain or the PHD finger. In addition, to investigate their molecular mechanism of action, MtING protein–protein interactors need to be identified and characterized.

## CONCLUSION AND OUTLOOK

Taken together, the phenotypes of the *Mting2* mutants suggest an important, non‐redundant role of *MtING2* in plant growth and flowering, which may involve an epigenetic mechanism. In contrast, *MtING1* appears to be redundant, in a wild‐type background, for the growth and flowering time phenotypes we observed, because *Mting1* mutants develop and flower normally. Further analysis by generating *Mting1 Mting2* double mutants is needed to identify their specific and redundant roles. In addition, MtING interactions with other proteins, including histone H3K4 methyltransferases, chromatin complexes, and histone marks, can be further pursued in the future. For example, MtINGs also bind H3K4me2 *in vitro* and H3K4me2 was recently shown to correlate with reduced transcript levels in rice (*Oryza sativa*), indicating that it functions as a repressive epigenetic mark (Liu et al., 2019). Furthermore, mass spectrometry and other assays have shown that AtING2 forms a complex with the Arabidopsis acetyl transferase transcriptional co‐activator NuA4 (Bieluszewski et al., 2015; Bieluszewski et al., 2022; Espinosa‐Cores et al., 2020), consistent with the ING domain of the yeast ING (YNG2) forming part of the structural complex of yeast NuA4 (Xu et al., 2016). Thus, MtING2 protein interactors should be identified *in planta* to further analyze how MtING2 regulates gene expression and chromatin modifications.

Our characterization of *MtING2* function in Medicago underscores the important role of INGs across eukaryotes, where in humans, their loss of function is linked to various diseases and developmental disorders (Russell et al.,^2006^). This study lays the groundwork for studying candidate genes that may be regulated by *MtING2* in control of flowering and plant development in Medicago. Overall, this study has provided evidence from the analysis of mutants of the physiological role of an *ING‐like* gene in plants, providing an impetus for analyzing the function of *ING* genes in other plants.

## EXPERIMENTAL PROCEDURES

### General bioinformatics

Medicago gene identifiers MtING2 (Medtr7g085450) and MtING1 (Medtr1g046460) were obtained from the ‘Jemalong A17’ accession in the *Medicago truncatula* Genome Database (Mt4.0v2; http://blast.jcvi.org/Medicago‐Blast/index.cgi) (Tang et al., 2014; Altschul et al., 1997). The *M. truncatula* wild type R108 genome assembly (v1.0 http://blast.jcvi.org/Medicago‐Blast/index.cgi) (Moll et al., 2017) was used to clone the R108 sequences and to aid primer design. Primers were designed using the Primer3 plugin (Untergasser et al., 2012) in the Geneious software package (v8.0 or later) (Kearse et al., 2012). Arabidopsis gene identifiers were obtained from TAIR (http://www.arabidopsis.org/). Other legume sequences were obtained from the Legume information system (Dash et al., 2016), specifically those belonging to annotated gene families legfed_v1_0.L_BNLHCY and legfed_v1_0.L_FY4ZFK. These were supplemented with additional representative sequences from other angiosperms present in the Phytozome databases (Goodstein et al., 2012) annotated as protein homologs of ING1 and ING2. Sequences were aligned using MAFFT (v7.475) (Katoh et al., 2005) using the L‐INS‐i method and a maximum‐likelihood phylogeny was created with FastTree (v2.1.11) (Price et al., 2010) using default parameters. Conserved protein functional domains were predicted by Pfam (https://pfam.xfam.org/) (Mistry et al., 2021) and SMART (http://smart.embl‐heidelberg.de/) (Letunic et al., 2021). *In silico* analysis of publicly available normalized RNA‐seq data in Figure 1 was conducted using the MedicMine *Medicago truncatula* Genome Database (http://medicmine.jcvi.org/medicmine/begin.do) (Krishnakumar et al., 2015). ShinyGO 0.76 (http://bioinformatics.sdstate.edu/go/) (Ge et al., 2020) or AgriGO v2.0 (Tian et al., 2017) was used for GO analysis of the significantly overrepresented genes in the biological processes, cellular components, and molecular functions of candidate genes identified from RNA‐seq. Gene identifiers were from Mt4.0v2, and *P*‐values (Fisher) were corrected for multiple testing adjustments (false discovery rate [FDR]). GO terms with *P* < 0.05 were considered significant.

### Plant materials, growth conditions, and flowering time

Wild‐type *M. truncatula* (Medicago) R108_C3 (R108) (Trinh et al., 1998) and *A. thaliana* Col plants were used in this study. Seeds of the tobacco retrotransposon *Tnt1* mutant allele for *MtING2* in the wild‐type R108 background, *Mting2‐1* (NF1633), were obtained from the Noble Research Institute (Ardmore, OK, USA) (Tadege et al., 2009). Transgenic and gene‐edited lines were generated in this project. For the typical growth of Medicago plants, seeds were scarified in between two pieces of sandpaper (grade P160), sterilized in chlorine solution (Millipore, USA) for 10 min, and germinated overnight at 15°C in the dark. Germinated seeds with or without prior vernalization treatment (3 weeks at 4°C in moist filter paper) were planted in seed raising mix (Daltons Limited, NZ) and placed on sub‐irrigated rockwool mats watered with hydroponic nutrient medium (Gibeaut et al., 1997; without Na_2_SiO_3_) or transplanted after 11–14 days to 2‐L pots of soil mix consisting of 9 parts of potting mix (Daltons Limited, NZ), 3 parts of vermiculite (Pacific Growers Supplies Limited, NZ), and 1 part of number 2 sand (Daltons Limited, NZ). Plants were grown in controlled rooms and growth cabinets in LD (16 h light/8 h dark) or SD (8 h light/16 h dark) photoperiods at 22°C with a light intensity of 120–150 μmol m^−2^ sec^−1^. Flowering time was scored as days after planting and the number of nodes on the primary axis at the time the first floral bud was observed was recorded (Jaudal, Thomson, et al., 2020). The length of the primary shoot axis was measured from the monofoliate leaf node to the uppermost shoot apical bud (Jaudal, Wen, et al., 2020). The longest secondary axis that branched off from the primary shoot axis was also identified and its entire length was measured. Genotyping was done using gene‐ and *Tnt1*‐specific primers (Table S1).

### Generating transgenic Medicago and Arabidopsis plants

CRISPR/Cas9 *Mting1* and *Mting2* gene‐edited mutant plants were generated in this work. Six or seven single guide RNAs (sgRNA) were designed for *MtING1* or *MtING2*, respectively, using Geneious software (v2020.0.5) (Kearse et al., 2012) to target the R108 wild‐type CDSs. The sgRNAs (Table S1) were identified based on N(17)VVR(NGG)H target selection criteria and activity scoring by Doench et al. (2014) and checked for the absence of off‐target sites in the Medicago R108 genome (v1.0). A construct consisting of a polycistronic pre‐tRNA‐sgRNA scaffold (Xie et al., 2015) cassette placed downstream of the Medicago U6 promoter with *Hin*dIII restriction sites added on each end was commercially synthesized (GenScript, USA) and inserted into the pCBSG041 plasmid vector backbone with the *Cas9* gene driven by the CAMV 35S promoter. The plasmid was transformed into *Agrobacterium tumefaciens* strain EHA105 and used to transform leaf tissues of wild type R108. Independent T0 transformant plants were selected on phosphinothricin and regenerated as described previously (Cosson et al., 2006; Jaudal et al., 2018). Young plants were also sprayed with Basta herbicide (Bayer, Germany) to select for transgenic plants. The *MtING* genes were analyzed for gene edits by analyzing the DNA sequence of genomic DNA and cDNAs. T1 seed and those of subsequent generations were generated by self‐fertilization of parent plants. In additional analysis, the *MtING1* genomic sequence was confirmed to be unchanged from wild type R108 in the *Mting2* gene‐edited plants.

*35S:MtING2‐3XFLAG* Medicago plants were generated in this study. For cloning, *MtING2* full‐length R108 cDNA with 3× FLAG was cloned into the pB2GW7 plasmid vector (Karimi et al., 2002) using the Gateway cloning system (Thermo Fisher, USA). *Agrobacterium* EHA105 containing the construct was used to transform leaf tissues of wild‐type R108 and *Mting2‐1* heterozygous mutant plants. Transgenic plants overexpressing *MtING2* were selected as described above.

Transgenic Arabidopsis plants overexpressing *MtING1* (*35S:MtING1*) or *MtING2* (*35S:MtING2*) were generated by inserting the R108 cDNAs into pB2GW7 (Karimi et al., 2002) using the Gateway cloning system (Thermo Fisher, USA). *Agrobacterium* GV3101 containing the pB2GW7 vectors was transformed into wild‐type *A. thaliana* Col using floral dipping, and transgenic T1, T2, and T3 plants were selected by spraying with Basta herbicide (Bayer, Germany) and genotyping (Jaudal et al., 2015; Martinez‐Trujillo et al., 2004). Flowering time of Arabidopsis plants was measured as either the total number of days or the total number of rosette and cauline leaves when the first floral buds were seen.

Flowering time results for Medicago and Arabidopsis and shoot axis measurements for Medicago are shown as mean with 95% confidence intervals.

### qRT‐PCR analysis of gene expression

Leaf and shoot apices were harvested separately at Zeitgeber time 4 (4 h after dawn), unless otherwise specified. Frozen plant tissues were ground to a fine powder with a Geno/Grinder R 2010 (SPEX R SamplePrep, USA). Total RNA extraction, cDNA synthesis using an oligo dT primer, and qRT‐PCR (primers are listed in Table S1) were carried out as previously described (Laurie et al., 2011; Zhang et al., 2019). Gene expression relative to the reference gene *PROTEIN PHOSPHATASE 2A* (*PP2A*) (Medtr6g084690) was calculated based on the 2^−ΔΔCt^ method (Livak & Schmittgen, 2001) with modifications (Bookout & Mangelsdorf, 2003) and calibrated to the highest value as previously described (Jaudal et al., 2018) unless stated otherwise. Each data point is the mean of three biological replicates harvested in parallel, with each replicate consisting of a pool of tissues from three independent plants, unless indicated otherwise. The identity of the PCR amplicons was checked by DNA sequencing. Statistical testing to analyze significant differences in gene expression was performed using one‐way analysis of variance (α = 0.05).

### Western blot

Total protein was extracted from the leaves of 4‐week‐old wild‐type R108 and transgenic 35S:MtING2‐3xFLAG Medicago plants grown in VLD conditions using the trichloroacetic acid/acetone method as described previously (Isaacson et al., 2006). Protein was separated by SDS‐PAGE (20 μg per lane) and transferred onto 0.2‐μm nitrocellulose membrane (Whatman, UK). The membrane was blocked in Tris‐buffered saline + 0.1% Tween 20 with 5% low‐fat milk powder. MtING2‐3xFLAG protein was detected using 1 μg/μl of mouse monoclonal anti‐FLAG (Sigma, F1804) and 80 pg/μl of horseradish peroxidase‐conjugated goat anti‐mouse IgG (Jackson ImmunoResearch Laboratories, USA). Images were captured using an Amersham Imager 600 (General Electric, USA) with Pierce SuperSignal West Femto Substrate (Thermo Fisher, USA).

### RNA extraction and RNA‐seq

Germinated seeds from wild‐type R108 and *Mting2‐1* (F4, 1.5BC) mutant plants were transferred to half‐strength SH9 medium with half‐strength sucrose (Cosson et al., 2006) in tubs, vernalized at 4°C for 3 weeks, and then grown in LD conditions (VLD) (16 h light/8 h dark) in growth cabinets. Trifoliate leaves or shoot apices were harvested from 13–15‐day‐old plants at 4 h after dawn (ZT4). Three biological replicates per tissue type for each genotype were harvested. Total RNA was extracted using the RNeasy Plant Mini Kit (Qiagen, Germany) following the manufacturer's instructions. The quantity and quality of RNA samples were checked using a Bioanalyzer 2100 (Agilent Technologies, USA). The samples were then sent to Macrogen Inc. (South Korea) for sequencing. The 12 RNA‐seq libraries were prepared using the TruSeq Stranded mRNA kit (Illumina, USA). Paired‐end (PE) sequencing was done on an Illumina platform (NovaSeq6000, 150 bp) aimed to generate 100 million reads per sample.

### RNA‐seq analysis

The quality of the sequenced raw reads (FASTQ files) was assessed using FastQC (v0.11.7), with reports combined using MultiQC (v1.7). To remove residual adaptors and low‐quality sequences from the reads, the BBDuk tool in the BBTools suite was used (v37.54) (Bushnell, 2018). Reads were trimmed from the 3′ end where quality dropped below a PHRED score of 20 and any remaining reads under 36 bp in length were also excluded. Trimmed reads were mapped to the Mt4.0v2 transcriptome (Tang et al., 2014; Young et al., 2011) using Salmon (v0.8.2) (Patro et al., 2017). The resulting count tables, in which abundance is measured as transcripts per million, were then imported into R (R Core Team, 2018) using the tximport package (v1.12.0) (Soneson et al., 2015). Normalization and differential expression analysis visualization were performed using DESeq2 (v1.24.0) (Love et al., 2014). Differentially expressed transcripts were filtered out using the following criteria: adjusted *P*‐value ≤ 0.05 and ¦log_2_(fold change)¦ ≥1. Venn analysis was conducted using Venny (http://bioinfogp.cnb.csic.es/tools/venny) (Oliveros, 2007‐2015). Enrichment analyses were performed in R using the goseq package (v1.36.0) (Young et al., 2010). To provide more information about known and novel transcripts and alternatively spliced transcripts, quality‐trimmed reads from each sample were also mapped to the Medicago genome (Mt4.0v2) (Tang et al., 2014; Young et al., 2011) using STAR (v2.7.9) and adopting the two‐pass approach with default parameters (Dobin et al., 2013). The publicly available RNA‐seq data from shoot apices of segregating late‐flowering Medicago mutants (*Mtfda_30d* [30 days old], *Mtfta1_30d* [30 days old], *Mtfta1_63d* [63 days old], and the *Mtfdafta1_63d* double mutant [63 days old]) were downloaded from NCBI (PRJNA649443) (Cheng et al., 2020). The same bioinformatic packages and tools used in RNA‐seq analysis were used to obtain the DEGs from the late‐flowering mutants compared to the wild type, which were then compared with *Mting2‐1* DEGs to determine the genes that were regulated in the same way. The five‐way Venn diagrams were created using InteractiVenn (Heberle et al., 2015) to visualize and compare multiple DEG datasets of RNA‐seq from the late‐flowering Medicago mutants and *Mting2‐1* at the vegetative stage.

### ChIP‐seq

Seeds from wild type R108 and *Mting2‐1* (F4, 1.5BC) were scarified and sterilized in chlorine solution (Millipore, USA) for 45 min and germinated overnight at 15°C in the dark with gentle shaking. Germinated seeds were transferred to half‐strength SH9 medium with half‐strength sucrose (Cosson et al., 2006) in tubs, vernalized at 4°C for 3 weeks, and then grown in LD conditions (16 h light/8 h dark) in growth cabinets. One gram of whole aerial tissues was harvested from 14–17‐day‐old plants (approximately three‐leaf stage) at 4 h after dawn. Three biological replicates per tissue type for each genotype were harvested. ChIP assays were performed as described previously (Wu et al., 2018, modified from Gregis et al., 2009) with the following modifications. The plant tissues were crosslinked under vacuum for 15 min. Glycine (0.125 m) was added to stop the crosslinking reaction and washed by cold sterile water with 1 mm PMSF, blotted dry, snap‐frozen, and ground into fine powder in liquid N_2_. Chromatin was extracted and sonicated for 10 cycles (30 s on, 30 s off) using a Bioruptor® Pico sonication device (Diagenode, USA) to obtain a size range of 200–500 bp. Protein A (16‐661) (Millipore, USA) was washed once and resuspended in ChIP Dilution Buffer. The chromatin was pre‐cleared with protein A magnetic beads and then incubated overnight with 7 μl of monoclonal anti‐H3K4me3 antibody (05‐745R) (Millipore, USA), 50 μl magnetic bead suspension, and 500 μl ChIP Dilution Buffer with rotation at 4°C. The beads were collected using a magnetic rack, washed, and reverse crosslinked, and DNA was purified using the phenol–chloroform extraction method. In total, six ChIP‐seq libraries (three biological replicates from wild type R108 and three from the *Mting2‐1* mutant) were sent to Novogene (China) for ChIP‐seq library preparation and sequencing. Briefly, the DNA samples were quality‐checked and the DNA libraries were prepared using the NEBNext ChIP‐Seq Library Prep Kit for Illumina (NEB, USA). PE sequencing was done on an Illumina platform (NovaSeq 6000, 150 bp) aimed to generate 20 million PE reads (6 Gb raw data) per sample.

### ChIP‐seq analysis

All three biological replicates from wild type R108 and *Mting2‐1* were sequenced; however, only two biological replicates from each were analyzed further. ChIP‐seq datasets were aligned against the *M. truncatula* genome (Mt4.0v2) (Tang et al., 2014) using Bowtie2 with default parameters (Langmead & Salzberg, 2012). Only uniquely mapped reads were retained (Picard toolkit; https://broadinstitute.github.io/picard/faq.html) running MarkDuplicates with REMOVE_DUPLICATES = true. The mapped reads were then filtered based on mapping quality using samtools (−q 30) (Li, 2011; Li et al., 2009). ChIP binding peaks were called by MACS2 (Zhang et al., 2008). Only peaks detected by MACS2 and present in both biological replicates of wild type R108 and the *Mting2‐1* mutant were kept (using bedtools intersect; Quinlan & Hall, 2010). Peaks were defined as specific when there were no peaks detected in the same region between the wild type R108 and the *Mting2‐1* mutant. The reads per kilobase per million (RPKM) values and the heatmap for the ChIP‐seq datasets were computed using deepTools2 (Ramírez et al., 2016).

### ChIP‐qPCR

In an independent experiment, 1 g of whole aerial tissues was harvested from 14–17‐day‐old plants (approximately three‐leaf stage) at 4 h after dawn grown in agar tubs. Four biological replicates for each genotype were harvested, where each replicate consisted of a pool of aerial tissues from 17–25 plants. ChIP assays were performed as above. Three biological replicates from each genotype were immunoprecipitated using protein A (16‐661) (Millipore, USA) magnetic beads and monoclonal anti‐H3K4me3 antibody (05‐745R) (Millipore, USA). The remaining biological replicate from each genotype was immunoprecipitated using protein A magnetic beads and anti‐rabbit IgG (W4011) (Promega, USA) as negative background IgG control. The purified DNA was used to perform ChIP‐qPCR analysis using SYBR®Green and a QuantStudio™ 5 Real‐Time PCR System (ThermoFisher, USA). The *anthocyanin acyltransferase* gene was included as negative control. H3K4me3 enrichment on genomic regions was calculated as mean percent input of three biological replicates. The identity of the qPCR amplicons was checked by DNA sequencing. Primer sequences used are listed in Table S1.

### *In vitro* protein–histone peptides binding assay

The CDSs of the PHD fingers from Medicago *ING1* and *ING2*, MtING1_PHD_ (M180–K247) and MtING2_PHD_ (M196–Q263), were cloned into a modified pET‐49b‐MBP vector. Proteins were expressed in *Escherichia coli* LOBSTR (DE3) cells (Andersen et al., 2013). Cells were incubated at 37°C in ZYM 505 (Studier, 2005) + 100 μm ZnCl_2_ until 0.6 OD_600_, followed by overnight induction with 1 mm isopropyl β‐d‐1‐thiogalactopyranoside at 18°C. The MBP‐ING1_PHD_ and MBP‐ING2_PHD_ proteins were purified by affinity chromatography on amylose‐resin. The MBP tag was removed using 3C‐protease and the tag was separated from the PHD finger by size exclusion chromatography. The purified PHD domains were dialyzed into Biacore running buffer (300 mm NaCl, 10 mm Tris pH 8, 0.05% Tween 20, 0.1 mm Tris(2‐carboxyethyl)phosphine). Binding of purified ING proteins to modified histone peptides was investigated by surface plasmon resonance using a Biacore X100 (GE healthcare, USA). Histone 3 peptides, i.e., unmodified H3 (1–21) (AS‐61702), H3K4me1 (AS‐64355), H3K4me2 (AS‐64356), H3K4me3 (AS‐64357), and H4K9me3 (AS‐64360) (Anaspec, USA), with a C‐terminal biotin moiety were immobilized using a Biotin CAPture chip (28‐9202‐33) to achieve a Δresonance unit of approximately 10. The ING1_PHD_ or ING2_PHD_ protein was loaded onto the machine at a flow rate of 10 μl/min for each peptide at concentrations between 0.39 and 400 μm. Data were analyzed using the Biacore X100 evaluation software with an equilibrium binding isotherm model.

## CONFLICT OF INTEREST

The authors declare no competing interests.

## Supporting information


**Figure S1.** *MtING2* is broadly expressed in Medicago wild type R108 through developmental time courses.

**Figure S2.** MtING1 and MtING2 do not appear to interact with each other or subunits of a histone deacetylase complex in a yeast two‐hybrid assay.

**Figure S3.** Plants carrying mutations in *MtING1* generated by CRISPR/Cas9 gene editing develop and flower similarly to wild type.

**Figure S4.** Relative expression of *MtING2* in 68‐day‐old R108 and *Mting2‐1* plants in VLD conditions.

**Figure S5.** Overexpression of *MtING2* in wild type R108 and the *Mting2‐1* mutant.

**Figure S6.** qRT‐PCR on selected genes that were differentially expressed in RNA‐seq.

**Figure S7.** H3K4me3 level in the *Mting2‐1* mutant and wild type R108.

**Figure S8.** Analysis of H3K4me3 peaks on the genes differentially expressed in the apex or leaf of *Mting2‐1* compared to wild type R108.

**Table S1.** List of primers and guides.

**Table S2.** Description of *Mting1* and *Mting2* gene‐edited lines.

**Table S3.** DEGs in apex or leaf of wild type R108 and the *Mting2‐1* mutant, shortlist of candidate DEGs, and comparison of DEGs with those from other late‐flowering mutants.

**Table S4.** Gene Ontology (GO) analysis of DEGs in *Mting2‐1* relative to wild type R108.

**Table S5.** H3K4me3 peaks identified in the *Mting2‐1* mutant or wild type R108. Genic regions and DEGs enriched with H3K4me3 peaks in the *Mting2‐1* mutant.

**Table S6.** Lists of DEGs from apex or leaf of the *Mting2‐1* mutant overlapped with *Mting2‐1*‐specific H3K4me3 peaks compared with wild type R108.

## Data Availability

RNA‐seq raw data are available at GEO (accession number GSE189301). ChIP‐seq raw data are available at GEO (accession number GSE189617).
