# Supplementary figures and images for: MtING2 encodes an ING domain PHD finger protein which affects Medicago growth, flowering, global patterns of H3K4me3, and gene expression
Source: Plant J. 2022 Oct 17;112(4):1029–50. doi: 10.1111/tpj.15994 (PMC9828230; doi:10.1111/tpj.15994)

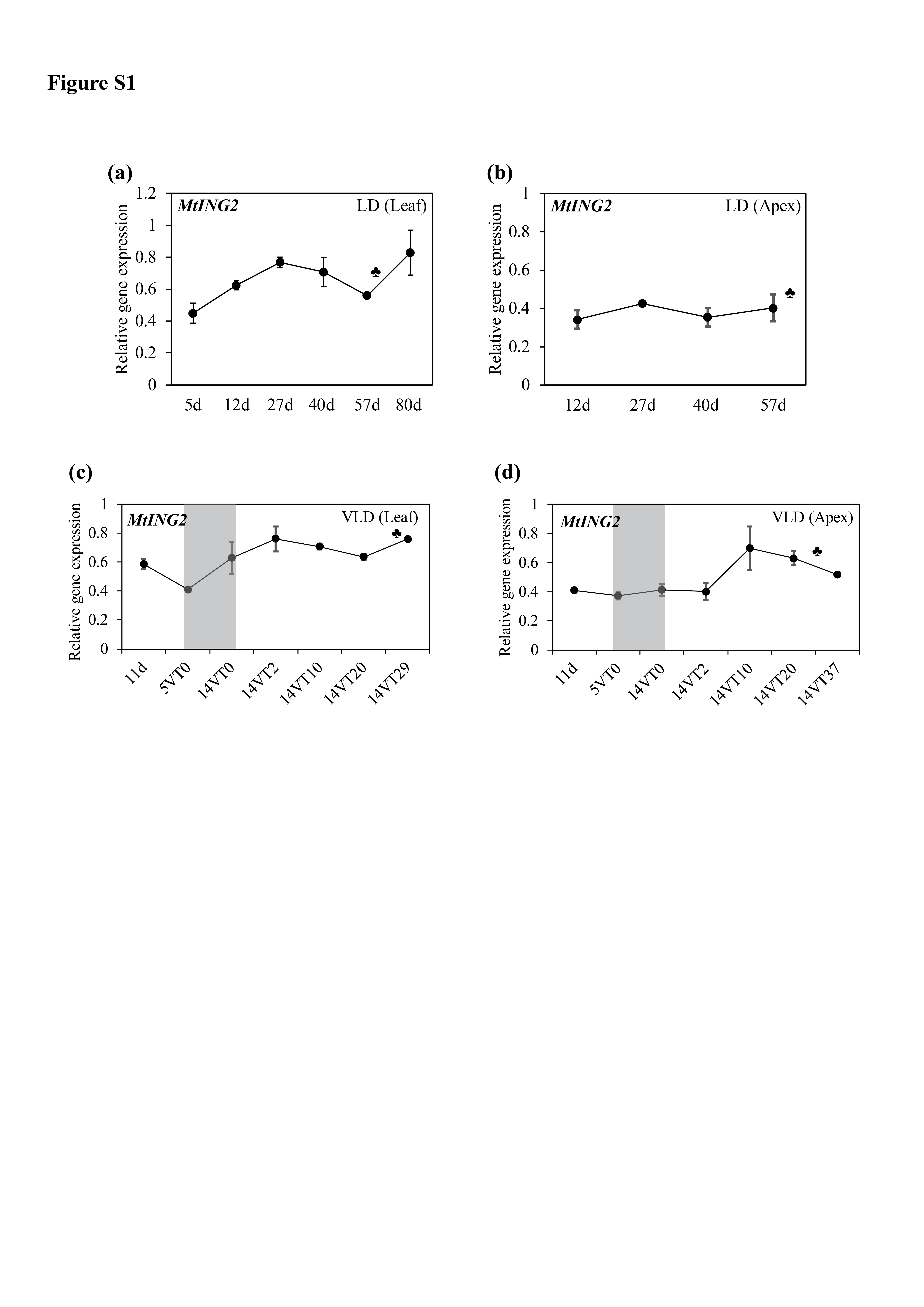

Supplement: Supplementary file 1 — Figure S1. MtING2 is broadly expressed in Medicago wild type R108 through developmental time courses. [file TPJ-112-1029-s007.jpg]

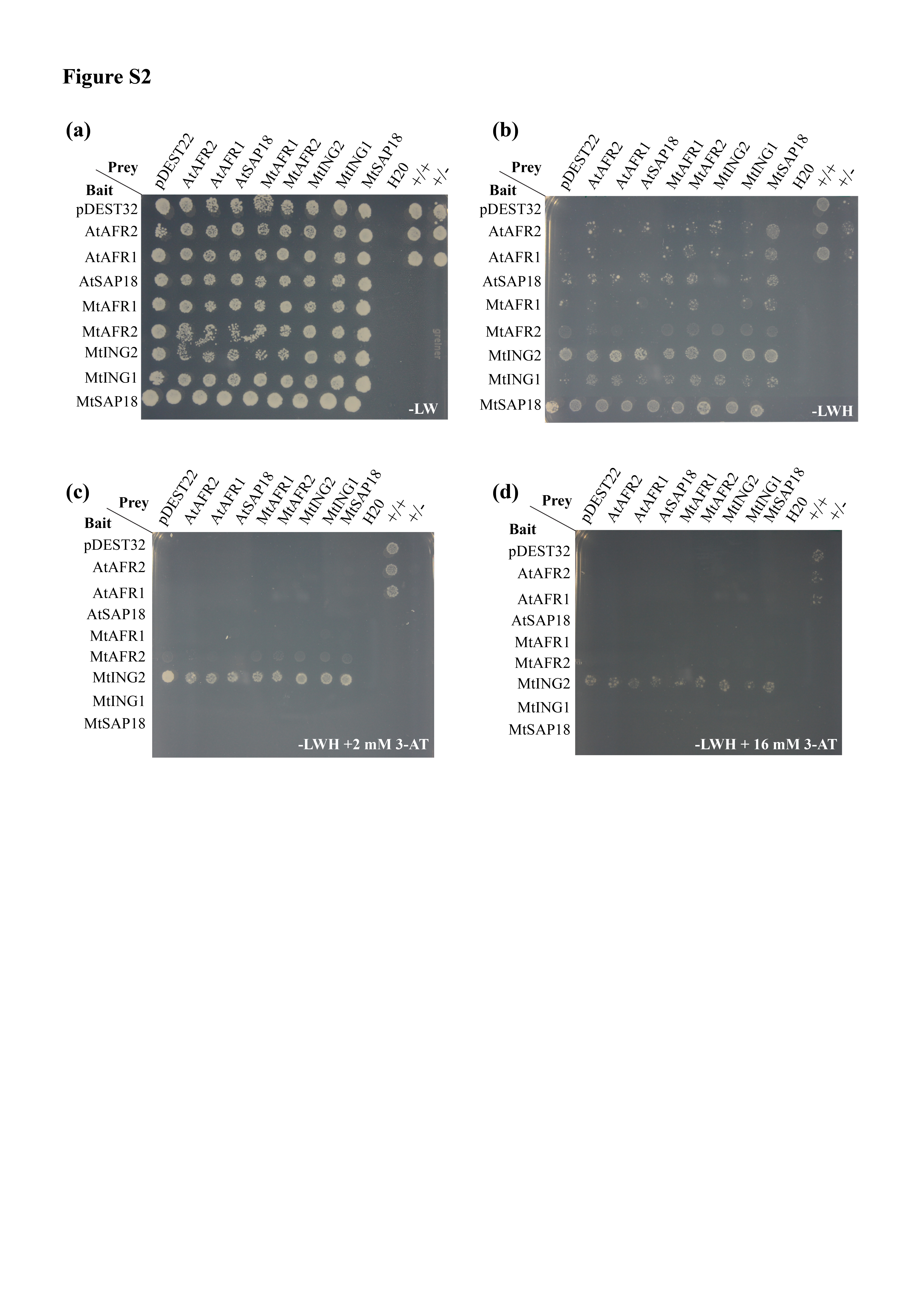

Supplement: Supplementary file 2 — Figure S2. MtING1 and MtING2 do not appear to interact with each other or subunits of a histone deacetylase complex in a yeast two‐hybrid assay. [file TPJ-112-1029-s006.jpg]

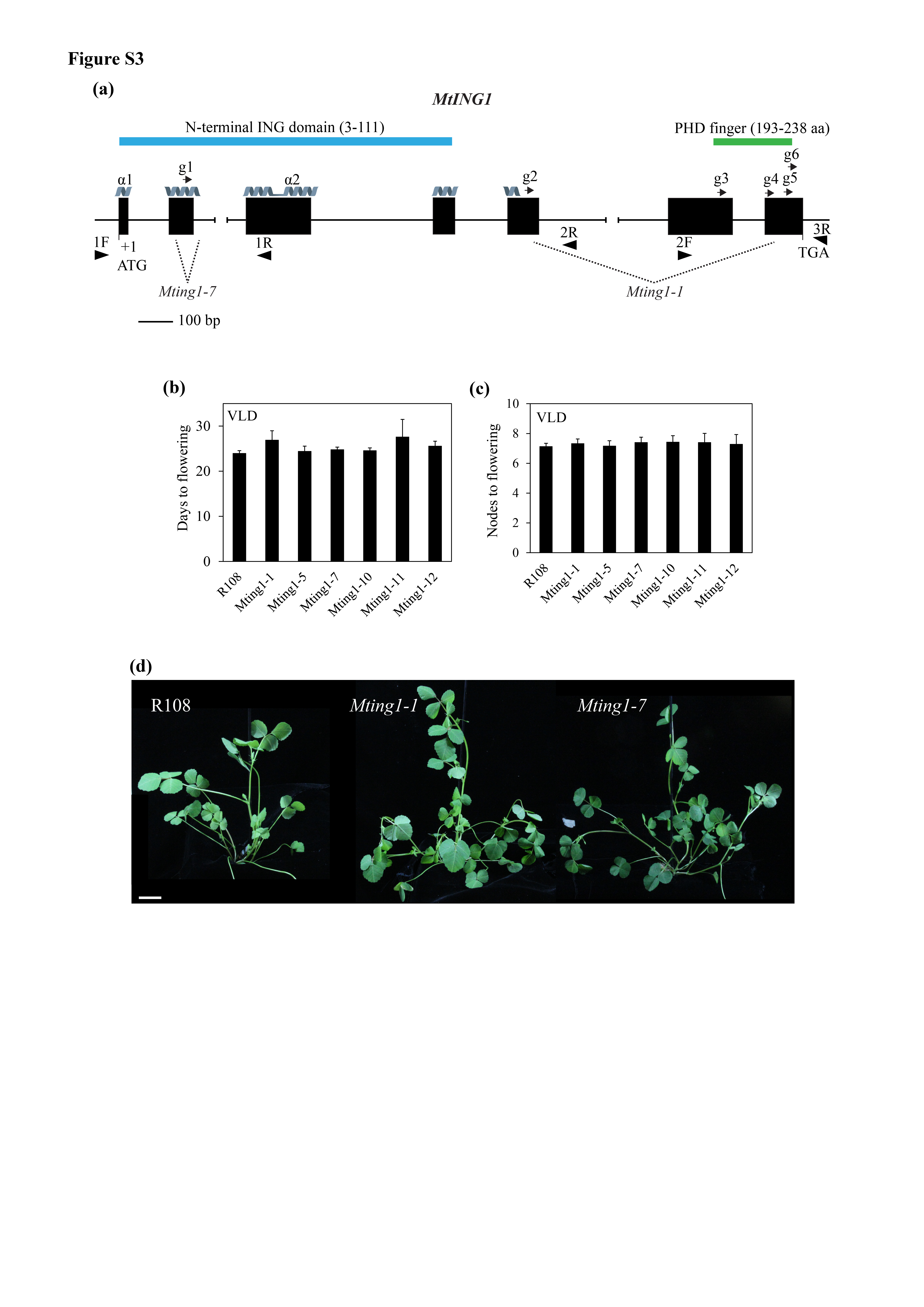

Supplement: Supplementary file 3 — Figure S3. Plants carrying mutations in MtING1 generated by CRISPR/Cas9 gene editing develop and flower similarly to wild type. [file TPJ-112-1029-s011.jpg]

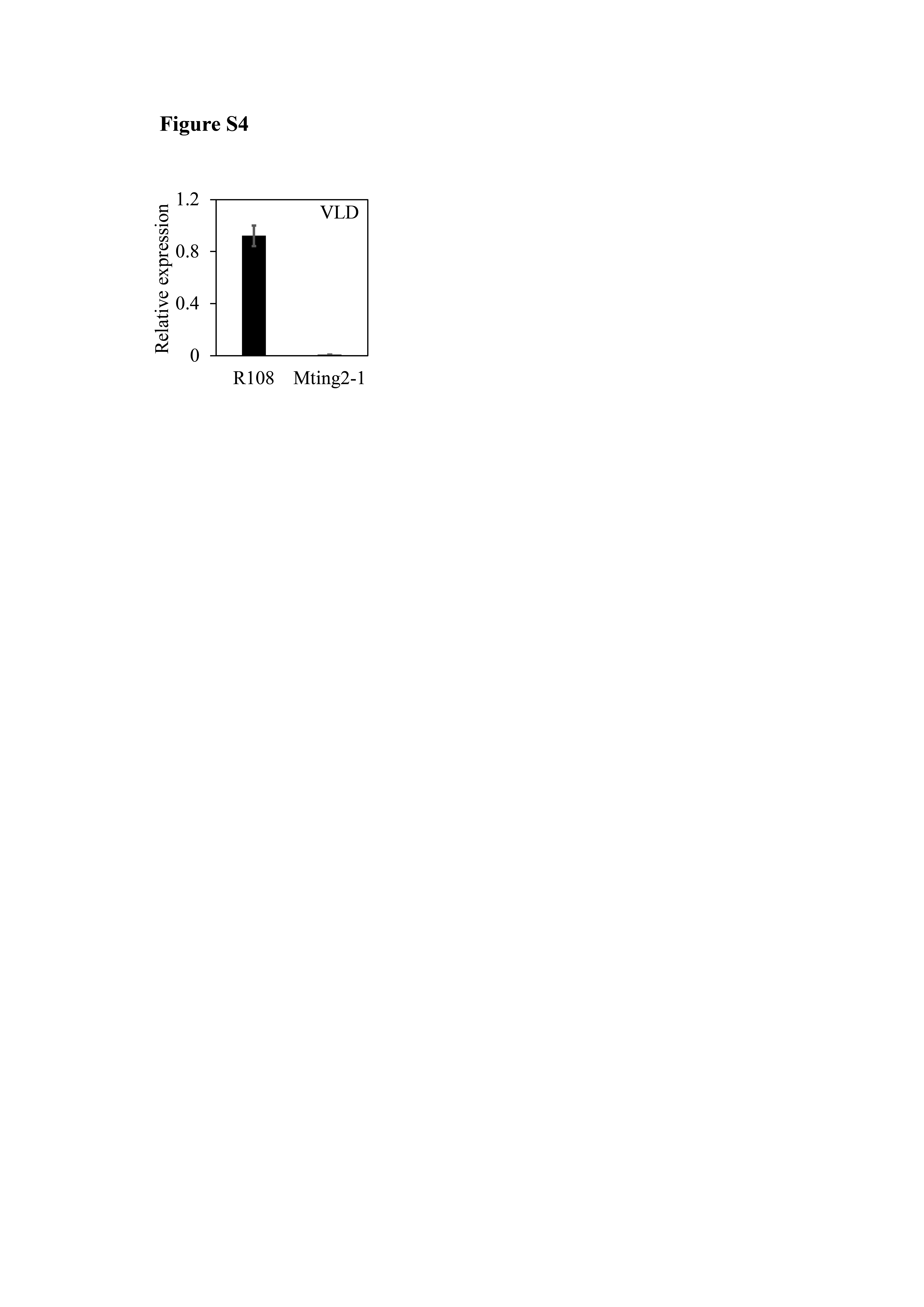

Supplement: Supplementary file 4 — Figure S4. Relative expression of MtING2 in 68‐day‐old R108 and Mting2‐1 plants in VLD conditions. [file TPJ-112-1029-s014.jpg]

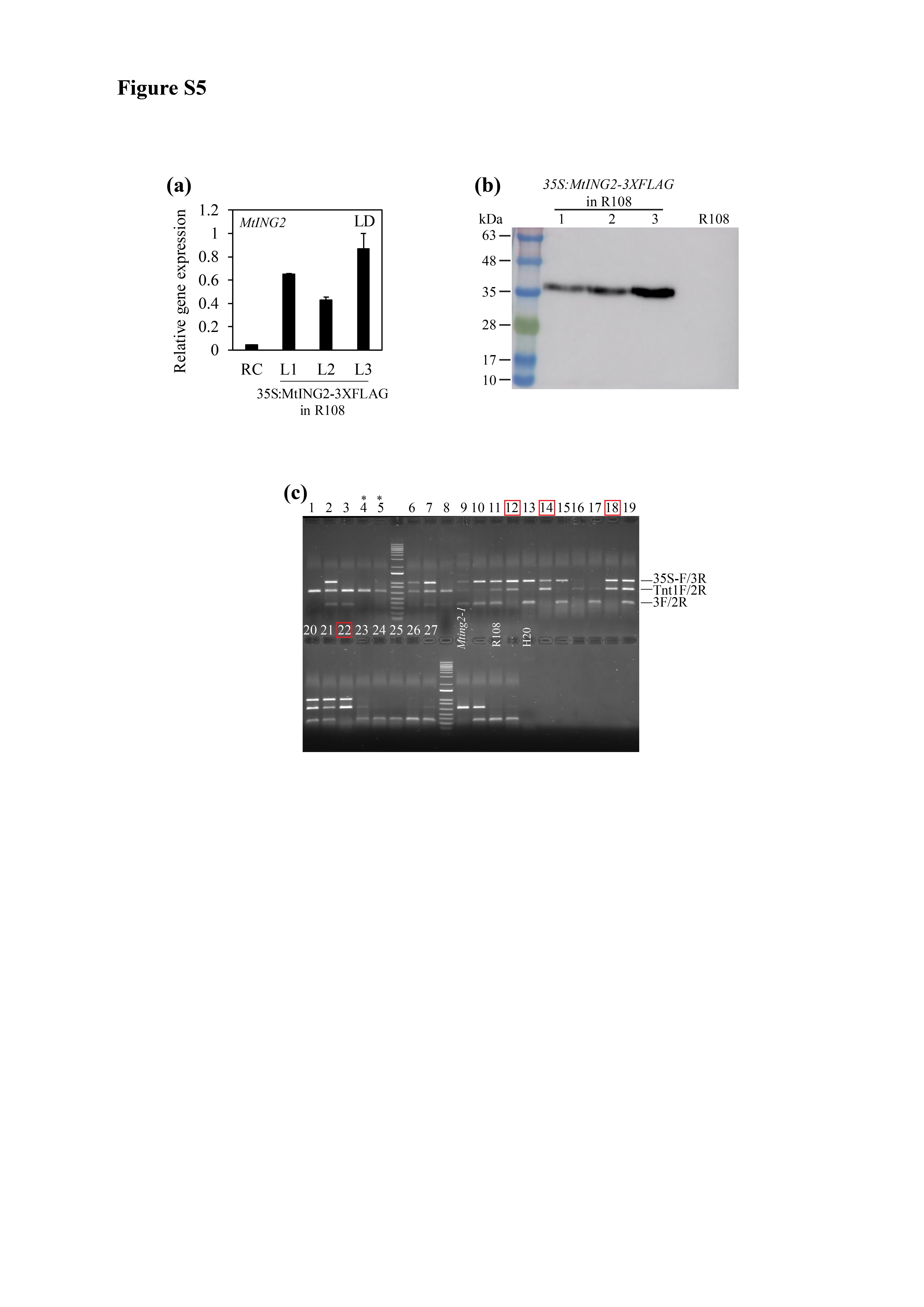

Supplement: Supplementary file 5 — Figure S5. Overexpression of MtING2 in wild type R108 and the Mting2‐1 mutant. [file TPJ-112-1029-s001.jpg]

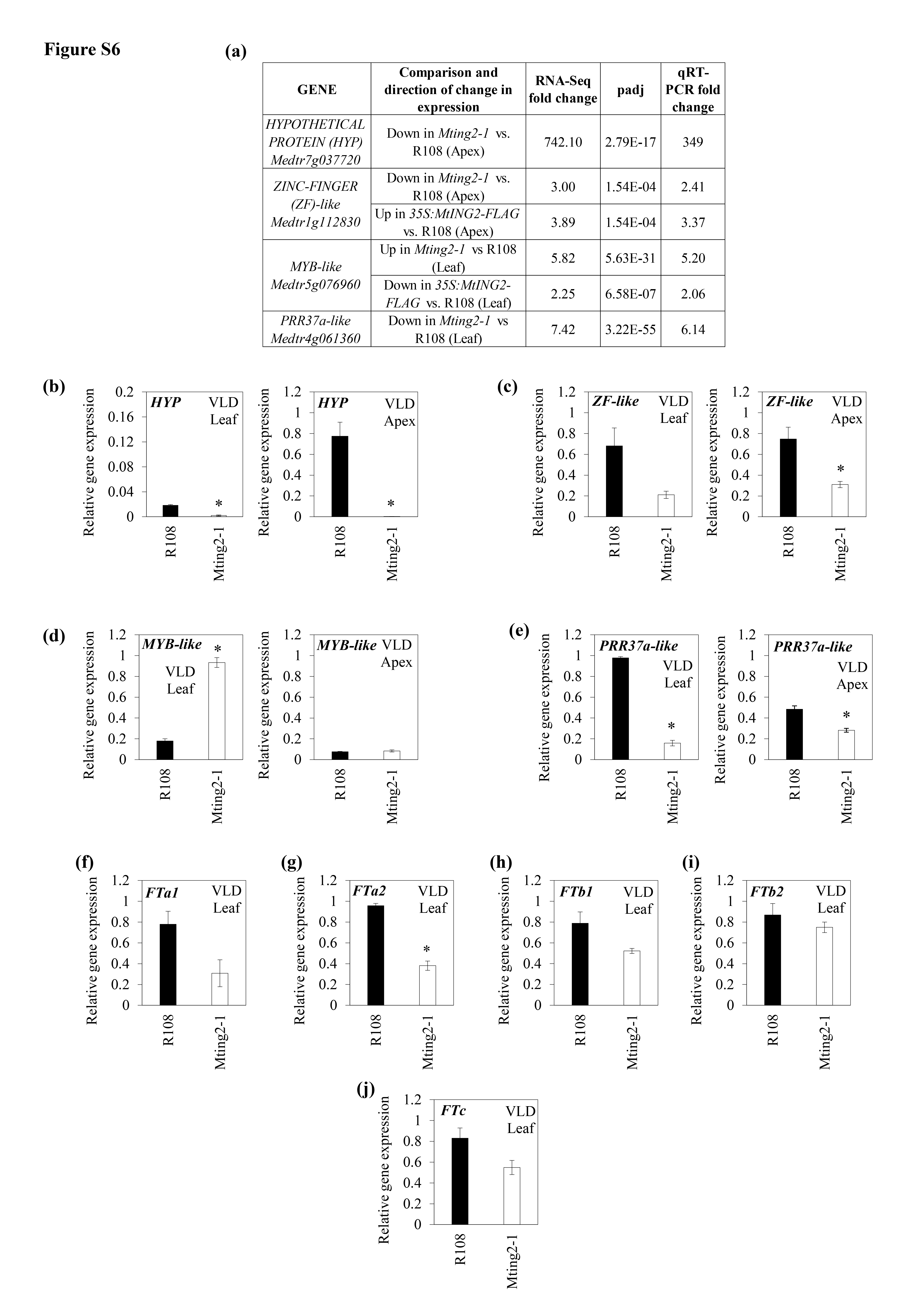

Supplement: Supplementary file 6 — Figure S6. qRT‐PCR on selected genes that were differentially expressed in RNA‐seq. [file TPJ-112-1029-s003.jpg]

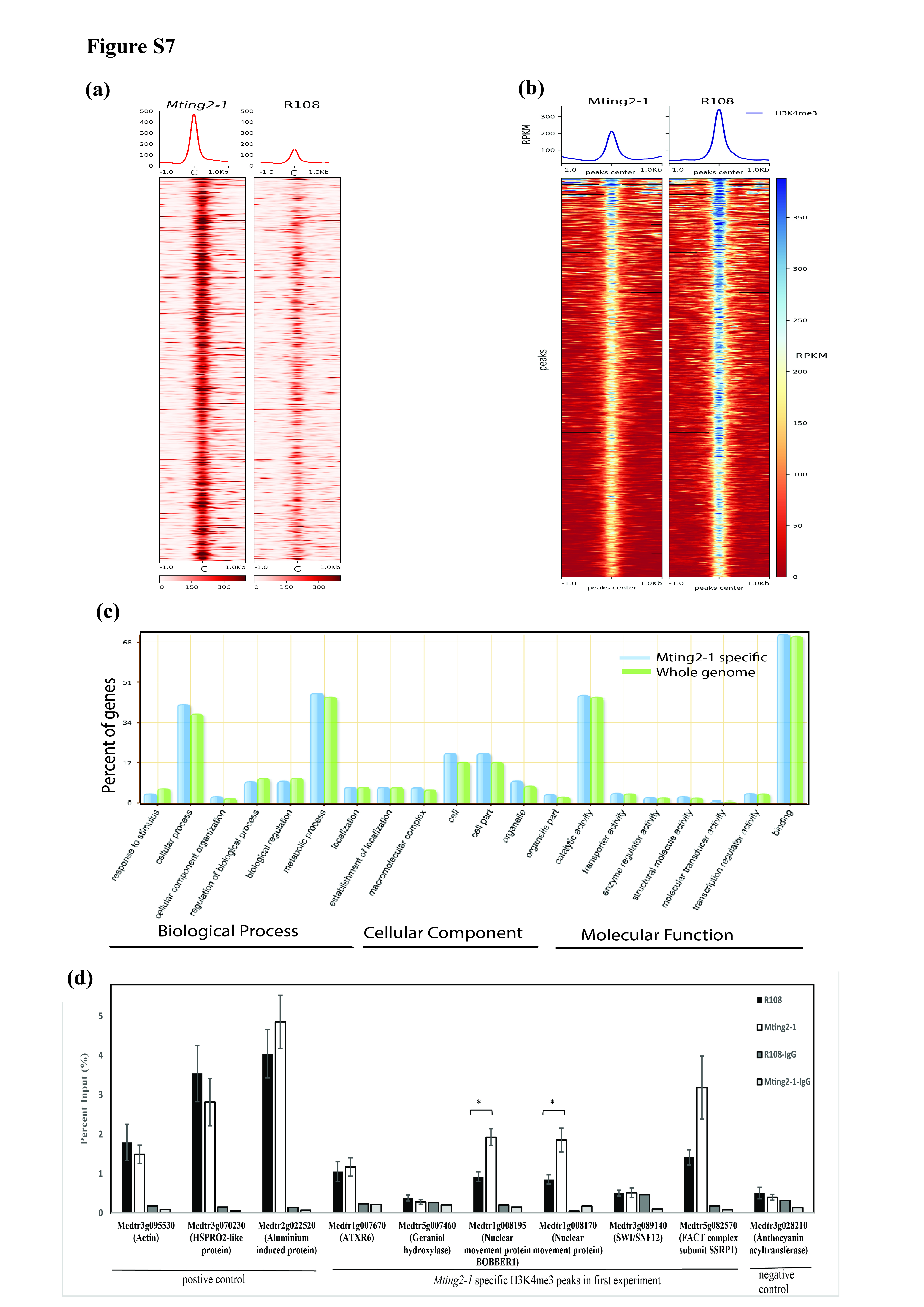

Supplement: Supplementary file 7 — Figure S7. H3K4me3 level in the Mting2‐1 mutant and wild type R108. [file TPJ-112-1029-s009.jpg]

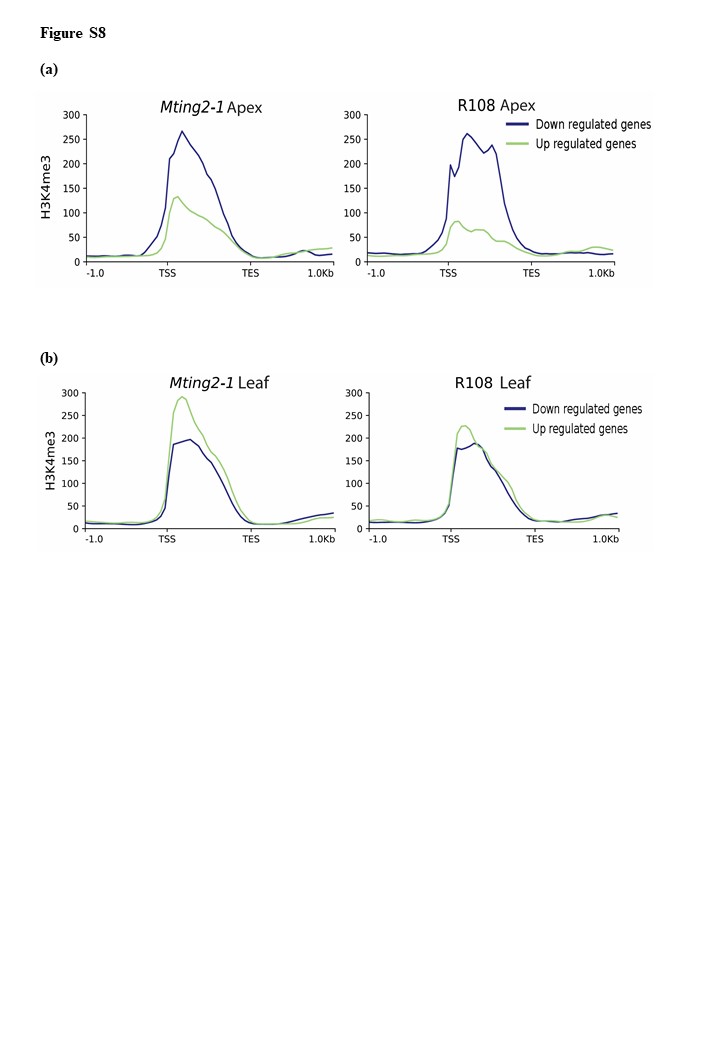

Supplement: Supplementary file 8 — Figure S8. Analysis of H3K4me3 peaks on the genes differentially expressed in the apex or leaf of Mting2‐1 compared to wild type R108. [file TPJ-112-1029-s010.jpg]
